# Supplementary material for: External validation of the improving partial risk adjustment in surgery (PRAIS-2) model for 30-day mortality after paediatric cardiac surgery
Source: BMJ Open. 2020 Nov 27;10(11):e039236. doi: 10.1136/bmjopen-2020-039236 (PMC7703410; doi:10.1136/bmjopen-2020-039236)
Supplement: Supplementary data [file bmjopen-2020-039236supp001.pdf]

Supplementary Table 1 Procedures with missing data on PRAIS2 compared with procedures with complete data

	**Non-missing**	**PRAIS2 Missing**
**n**	4373	371
**Age (median, IQR)**	2.55(4.02)	3.39(4.74)
**Diagnoses group n (%)** [a]		
GROUP 1	332(7.6)	51(13.7)
GROUP 2	506(11.6)	46(12.4)
GROUP 3	339(7.8)	27(7.3)
GROUP 4	672(15.4)	33(8.9)
GROUP 5	349(8.0)	36(9.7)
GROUP 6	341(7.8)	52(14.0)
GROUP 7	615(14.1)	21(5.7)
GROUP 8	452(10.3)	18(4.9)
GROUP 9	47(1.1)	1(0.3)
GROUP 10	165(3.8)	6(1.6)
GROUP 11	555(12.7)	21(5.7)
GROUP NA	0(0.0)	59(15.9)
**Procedure Group n (%)** [a]		
GROUP 1	60(1.4)	0(0.0)
GROUP 2	73(1.7)	0(0.0)
GROUP 3	237(5.4)	0(0.0)
GROUP 4	316(7.2)	13(3.5)
GROUP 5	811(18.5)	7(1.9)
GROUP 6	381(8.7)	5(1.3)
GROUP 7	116(2.7)	2(0.5)
GROUP 8	312(7.1)	3(0.8)
GROUP 9	62(1.4)	0(0.0)
GROUP 10	141(3.2)	0(0.0)
GROUP 11	112(2.6)	1(0.3)
GROUP 12	146(3.3)	0(0.0)

GROUP 13	258(5.9)	1(0.3)
GROUP 14	136(3.1)	2(0.5)
GROUP 15	636(14.5)	5(1.3)
GROUP 20	576(13.2)	9(2.4)
GROUP NA	0(0.0)	323(87.1)
**Bypass n (%)**	3175(72.6)	51(13.7)
**Weight (mean, SD)**	11.54(13.86)	14.16(16.78)
**UVH category, n (%)**		
No	3753(85.8)	274(73.9)
Yes	620(14.2)	73(19.7)
NA	0(0.0)	24(6.5)
**Severity of illness[b], n (%)**		
No	4065(93.0)	271(73.0)
Yes	308(7.0)	85(22.9)
NA	0(0.0)	15(4.0)
**Acquired comorbidity[b], n (%)**		
No	4063(92.9)	299(80.6)
Yes	310(7.1)	57(15.4)
NA	0(0.0)	15(4.0)
**Additional cardiac risk factors[b], n (%)**		
No	4181(95.6)	324(87.3)
Yes	192(4.4)	32(8.6)
NA	0(0.0)	15(4.0)
**Congenital comorbidity[b], n (%)**		
No	3583(81.9)	280(75.5)
Yes	790(18.1)	76(20.5)
Na	0(0.0)	15(4.0)
**30-day mortality, n (%)**	111(2.5)	31(8.4)

[a] as the diagnoses groups and procedure groups have lengthy text details, we have not provided them in the table; they can be found in Supplementary Table 5.

[b] Definitions of variables are given in Supplementary Table 5
