# Supplementary material for: External validation of the improving partial risk adjustment in surgery (PRAIS-2) model for 30-day mortality after paediatric cardiac surgery
Source: BMJ Open. 2020 Nov 27;10(11):e039236. doi: 10.1136/bmjopen-2020-039236 (PMC7703410; doi:10.1136/bmjopen-2020-039236)
Supplement: Supplementary data [file bmjopen-2020-039236supp004.pdf]

Supplementary table 4 Procedures with missing data on PRAIS2 compared with procedures with complete data in the Overlapping Cohort

	Non-missing	Missing
**n**	1824	180
**Age (mean, SD)**	2.43(3.93)	3.26(4.71)
**Diagnoses group n (%)** [a]		
GROUP 1	154(8.4)	24(13.3)
GROUP 2	194(10.6)	29(16.1)
GROUP 3	125(6.9)	8(4.4)
GROUP 4	222(12.2)	12(6.7)
GROUP 5	123(6.7)	7(3.9)
GROUP 6	100(5.5)	17(9.4)
GROUP 7	312(17.1)	16(8.9)
GROUP 8	192(10.5)	12(6.7)
GROUP 9	25(1.4)	1(0.6)
GROUP 10	95(5.2)	4(2.2)
GROUP 11	282(15.5)	14(7.8)
GROUP NA	0(0.0)	36(20.0)
**Procedure Group n (%)** [a]		
GROUP 1	28(1.5)	0(0.0)
GROUP 2	33(1.8)	0(0.0)
GROUP 3	84(4.6)	0(0.0)
GROUP 4	114(6.2)	6(3.3)
GROUP 5	344(18.9)	6(3.3)
GROUP 6	180(9.9)	3(1.7)
GROUP 7	50(2.7)	1(0.6)
GROUP 8	126(6.9)	3(1.7)
GROUP 9	26(1.4)	0(0.0)
GROUP 10	70(3.8)	0(0.0)
GROUP 11	57(3.1)	0(0.0)

GROUP 12	50(2.7)	0(0.0)
GROUP 13	103(5.6)	0(0.0)
GROUP 14	55(3.0)	2(1.1)
GROUP 15	275(15.1)	2(1.1)
GROUP 20	229(12.6)	5(2.8)
GROUP NA	0(0.0)	152(84.4)
**Bypass n (%)**	1361(74.6)	32(17.8)
**Weight (mean, SD)**	11.23(13.92)	13.99(17.63)
**UVH category, n (%)**		
No	1566(85.9)	128(71.1)
Yes	258(14.1)	39(21.7)
NA	0(0.0)	13(7.2)
**Severity of illness[b], n (%)**		
No	1738(95.3)	163(90.6)
Yes	86(4.7)	11(6.1)
NA	0(0.0)	6(3.3)
**Acquired comorbidity[b], n (%)**		
No	1730(94.8)	160(88.9)
Yes	94(5.2)	14(7.8)
NA	0(0.0)	6(3.3)
**Additional cardiac risk factors[b], n (%)**		
No	1772(97.1)	163(90.6)
Yes	52(2.9)	11(6.1)
NA	0(0.0)	6(3.3)
**Congenital comorbidity[b], n (%)**		
No	1550(85.0)	146(81.1)
Yes	274(15.0)	28(15.6)
NA	0(0.0)	6(3.3)
**30-day mortality, n (%)**	56(3.1)	15(8.3)

[a] as the diagnoses groups and procedure groups have lengthy text details, we have not provided them in the table; they can be found in Supplementary Table 5.

[b] Definitions of variables are given in Supplementary Table 5
