# Supplementary material for: External validation of the improving partial risk adjustment in surgery (PRAIS-2) model for 30-day mortality after paediatric cardiac surgery
Source: BMJ Open. 2020 Nov 27;10(11):e039236. doi: 10.1136/bmjopen-2020-039236 (PMC7703410; doi:10.1136/bmjopen-2020-039236)
Supplement: Supplementary data [file bmjopen-2020-039236supp003.pdf]

Supplementary table 3 Procedures with missing data on PRAIS2 compared with procedures with complete data in the Cohort 2

	Non-missing	Missing
n	1197	133
**Age (mean, SD)**	2.91(4.22)	3.49(4.76)
**Diagnoses group n (%)** [a]		
GROUP 1	107(8.9)	25(18.8)
GROUP 2	163(13.6)	12(9.0)
GROUP 3	103(8.6)	14(10.5)
GROUP 4	232(19.4)	16(12.0)
GROUP 5	122(10.2)	23(17.3)
GROUP 6	137(11.4)	26(19.5)
GROUP 7	127(10.6)	4(3.0)
GROUP 8	105(8.8)	3(2.3)
GROUP 9	2(0.2)	0(0.0)
GROUP 10	14(1.2)	1(0.8)
GROUP 11	85(7.1)	5(3.8)
GROUP NA	0(0.0)	4(3.0)
**Procedure Group n (%)** [a]		
GROUP 1	31(2.6)	0(0.0)
GROUP 2	11(0.9)	0(0.0)
GROUP 3	46(3.8)	0(0.0)
GROUP 4	103(8.6)	0(0.0)
GROUP 5	228(19.0)	0(0.0)
GROUP 6	53(4.4)	0(0.0)
GROUP 7	45(3.8)	0(0.0)
GROUP 8	100(8.4)	0(0.0)
GROUP 9	16(1.3)	0(0.0)
GROUP 10	35(2.9)	0(0.0)
GROUP 11	19(1.6)	0(0.0)
GROUP 12	64(5.3)	0(0.0)
GROUP 13	68(5.7)	0(0.0)
GROUP 14	43(3.6)	0(0.0)

GROUP 15	144(12.0)	0(0.0)
GROUP 20	191(16.0)	0(0.0)
GROUP NA	0(0.0)	133(100.0)
**Bypass n (%)**	911(76.1)	0(0.0)
**Weight (mean, SD)**	12.57(13.89)	14.31(15.94)
**UVH category, n (%)**		
GROUP 0	978(81.7)	98(73.7)
GROUP 1.2	219(18.3)	31(23.3)
GROUP NA.2	0(0.0)	4(3.0)
**Severity of illness [b], n (%)**		
Yes	222(18.5)	74(55.6)
**Acquired comorbidity [b], n (%)**		
Yes	207(17.3)	43(32.3)
**Additional cardiac risk factors [b], n (%)**		
yes	82(6.9)	17(12.8)
**Congenital comorbidity [b], n (%)**	343(28.7)	41(30.8)
**30-day mortality, n (%)**	23(1.9)	14(10.5)

[a] as the diagnoses groups and procedure groups have lengthy text details, we have not provided them in the table; they can be found in Supplementary Table 5.

[b] Definitions of variables are given in Supplementary Table 5
