# Supplementary material for: External validation of the improving partial risk adjustment in surgery (PRAIS-2) model for 30-day mortality after paediatric cardiac surgery
Source: BMJ Open. 2020 Nov 27;10(11):e039236. doi: 10.1136/bmjopen-2020-039236 (PMC7703410; doi:10.1136/bmjopen-2020-039236)
Supplement: Supplementary data [file bmjopen-2020-039236supp002.pdf]

Supplementary table 2 Procedures with missing data on PRAIS2 compared with procedures with complete data in the Cohort 1

	**Non-missing**	**Missing**
**n**	1352	58
**Age (median, IQR)**	2.39(3.93)	3.59(4.84)
**Diagnoses group n (%)** [a]		
GROUP 1	71(5.3)	2(3.4)
GROUP 2	149(11.0)	5(8.6)
GROUP 3	111(8.2)	5(8.6)
GROUP 4	218(16.1)	5(8.6)
GROUP 5	104(7.7)	6(10.3)
GROUP 6	104(7.7)	9(15.5)
GROUP 7	176(13.0)	1(1.7)
GROUP 8	155(11.5)	3(5.2)
GROUP 9	20(1.5)	0(0.0)
GROUP 10	56(4.1)	1(1.7)
GROUP 11	188(13.9)	2(3.4)
GROUP NA	0(0.0)	19(32.8)
**Procedure Group n (%)** [a]		
GROUP 1	1(0.1)	0(0.0)
GROUP 2	29(2.1)	0(0.0)
GROUP 3	107(7.9)	0(0.0)
GROUP 4	99(7.3)	7(12.1)
GROUP 5	239(17.7)	1(1.7)
GROUP 6	148(10.9)	2(3.4)
GROUP 7	21(1.6)	1(1.7)
GROUP 8	86(6.4)	0(0.0)
GROUP 9	20(1.5)	0(0.0)
GROUP 10	36(2.7)	0(0.0)
GROUP 11	36(2.7)	1(1.7)
GROUP 12	32(2.4)	0(0.0)

GROUP 13	87(6.4)	1(1.7)
GROUP 14	38(2.8)	0(0.0)
GROUP 15	217(16.1)	3(5.2)
GROUP 20	156(11.5)	4(6.9)
GROUP NA	0(0.0)	38(65.5)
**Bypass n (%)**	903(66.8)	19(32.8)
**Weight (mean, SD)**	11.04(13.73)	14.34(16.21)
**UVH category, n (%)**		
No	1209(89.4)	48(82.8)
Yes	143(10.6)	3(5.2)
NA	0(0.0)	7(12.1)
**Severity of illness[b], n (%)**		
NA	0(0.0)	9(15.5)
**Acquired comorbidity[b], n (%)**		
No	1343(99.3)	49(84.5)
Yes	9(0.7)	0(0.0)
NA	0(0.0)	9(15.5)
**Additional cardiac risk factors[b], n (%)**		
No	1294(95.7)	45(77.6)
Yes	58(4.3)	4(6.9)
NA	0(0.0)	9(15.5)
**Congenital comorbidity[b], n (%)**		
No	1179(87.2)	42(72.4)
Yes	173(12.8)	7(12.1)
NA	0(0.0)	9(15.5)
**30-day mortality, n (%)**	32(2.4)	2(3.4)

[a] as the diagnoses groups and procedure groups have lengthy text details, we have not provided them in the table; they can be found in Supplementary Table 5

[b] Definitions of variables are given in Supplementary Table 5
