# Supplementary material for: External validation of the improving partial risk adjustment in surgery (PRAIS-2) model for 30-day mortality after paediatric cardiac surgery
Source: BMJ Open. 2020 Nov 27;10(11):e039236. doi: 10.1136/bmjopen-2020-039236 (PMC7703410; doi:10.1136/bmjopen-2020-039236)
Supplement: Supplementary data [file bmjopen-2020-039236supp009.pdf]

Supplementary Equation 1. The final recalibrated model

$$Z = -0.229 - 0.439 \times \sqrt{agep} + 0.336 \times age - 1.808 \times \sqrt{weightp} + 0.088 \times weight + \sum coef$$
