# Supplementary material for: External validation of the improving partial risk adjustment in surgery (PRAIS-2) model for 30-day mortality after paediatric cardiac surgery
Source: BMJ Open. 2020 Nov 27;10(11):e039236. doi: 10.1136/bmjopen-2020-039236 (PMC7703410; doi:10.1136/bmjopen-2020-039236)
Supplement: Supplementary data [file bmjopen-2020-039236supp005.pdf]

Supplementary table 5. Glossary of variables included in the PRAIS2 model

Diagnoses Group	
1	HLHS; Truncus arteriosus; Pulmonary atresia and IVS
2	Functionally UVH; Pulmonary atresia and VSD
3	TGA þ VSD/DORV-TGA; Interrupted aortic arch
4	PDA
5	Miscellaneous primary congenital diagnosis; Tricuspid valve abnormality (including Ebstein’s); TAPVC; No diagnosis given
6	Acquire
7	AVSD; Fallot/DORV Fallot
8	Aortic valve stenosis (isolated); Mitral valve abnormality; Miscellaneous congenital terms
9	TGA + IVS
10	Aortic arch obstruction þ VSD/ASD; Pulmonary stenosis
11	Subaortic stenosis (isolated); Aortic regurgitation; VSD; ASD; Arrhythmia
Procedure diagnose group	

1	Norwood Procedure (Stage 1); HLHS hybrid approach
2	TAPVC repair + arterial shunt; Truncus and interruption repair; Truncus arteriosus repair; Interrupted aortic arch repair; Arterial switch + aortic arch obstruction repair (with-without VSD closure)
3	Arterial shunt
4	Repair of total anomalous pulmonary venous connection; Arterial switch + VSD closure; Isolated pulmonary artery band
5	PDA ligation (surgical)
6	Arterial switch (for isolated transposition); Isolated coarctation/hypoplastic aortic arch repair; Aortopulmonary window repair
7	Senning or Mustard procedure; Ross-Konno procedure; Mitral valve replacement; Pulmonary vein stenosis procedure; Pulmonary atresia VSD repair; Tetralogy with absent pulmonary valve repair; Unifocalization procedure (with/without shunt)
8	Heart transplant; Tricuspid valve replacement; Aortic valve repair; Pulmonary valve replacement; Aortic root replacement (not Ross);Cardiac conduit replacement; Isolated RV to PA conduit construction; Tricuspid valve repair
9	Multiple VSD closure; Atrioventricular septal defect and tetralogy repair; Cor triatriatum repair; Supravalvar aortic stenosis repair; Rastelli-REV procedure
10	Bidirectional cavopulmonary shunt
11	Atrioventricular septal defect (complete) repair
12	Fontan procedure
13	Aortic valve replacement: Ross; Subvalvar aortic stenosis repair; Mitral valve repair; Sinus venosus ASD and/or PAPVC repair
14	Atrioventricular septal defect (partial) repair; Tetralogy of Fallot–type DORV repair; Vascular ring procedure
15	Anomalous coronary artery repair; Aortic valve replacement: non-Ross; ASD repair; VSD repair
20	Cardiac no specific procedure

Acquired Comorbidity	Pulmonary embolism; Secondary systemic hypertension; Systemic hypertension; Primary (essential) systemic hypertension; Systemic hypertension due to aortic arch obstruction; Persistent pulmonary hypertension of the new-born; Necrotising enterocolitis; Meconium aspiration; Pre-procedural coagulation disorder; Pre-procedural renal failure or Pre-procedural renal failure requiring dialysis; Pre-procedural septicaemia; Pre-procedural neurological impairment; Preprocedural cerebral abnormality on imaging; Pre-procedural tracheostomy; Preprocedural seizures; Preprocedural respiratory syncytial virus (RSV) infection; Preprocedural necrotising enterocolitis: treated medically or surgically; Psychomotor developmental delay; Brain Abscess; Cerebrovascular accident (stroke); Anoxic-ischaemic encephalopathy; Hyperthyroidism; Diabetes mellitus or Diabetes mellitus requiring insulin; Meningitis; Kidney failure; Empyema; Lower respiratory tract infection; Lung disease; Asthma; Acquired bronchial disease; Airway disease; Diaphragm disorder: acquired; Diaphragm paralysis; Oesophageal disorder
Additional cardiac risk factor	Ventricular dyssynchrony; Arrhythmogenic right ventricular cardiomyopathy; Right or Left ventricular dysfunction; Ventricular myocardial noncompaction cardiomyopathy; Infectious myocarditis; Drug induced heart muscle disease; Trypanosomal myocarditis (Chagas' disease); Myocardial failure in end stage congenital heart disease; Heart muscle disease in cardiac rejection; Nutritional heart muscle disease; Heart muscle disease in infant of diabetic mother; Heart muscle disease in collagen vascular/ connective tissue disorder; Myocarditis; Ischaemic heart disease; Cardiomyopathy; Restrictive cardiomyopathy; Idiopathic restrictive cardiomyopathy; Endocardial fibroelastosis; Infiltrative cardiomyopathy; Hypertrophic cardiomyopathy; Dilated cardiomyopathy; Pulmonary arterial hypertension; Idiopathic (primary) pulmonary hypertension; Pulmonary vascular disease; Irreversible pulmonary vascular disease due to congenital heart disease (Eisenmenger Syndrome); Secondary pulmonary hypertension; Pulmonary hypertension due to congenital systemic-to-pulmonary shunt; Elevated lung resistance for biventricular repair (> 6 Wood units); Elevated lung resistance for heart transplant (> 4 Wood units); Elevated lung resistance for univentricular repair (> 2 Wood units); Transient myocardial ischaemia; Myocardial infarction; Acute myocardial infarction; Pre-procedural pulmonary hypertension; Preprocedural myocardial infarction; Preprocedural pulmonary hypertension (pulmonary pressure more than or equal to systemic pressure): echo data or catheter data; Residual pulmonary hypertension after relief of L to R shunt

Congenital comorbidity	Visceral heterotaxy (abnormal arrangement thoraco-abdominal organs); Position or morphology of thoraco-abdominal organs abnormal; Lung anomaly; Functionally congenital single lung; Tracheobronchial anomaly; Intestines malrotated; Hereditary disorder associated with heart disease; Trisomy 18 - Edwards syndrome; Trisomy 13 - Pataus syndrome; 45XO - Turners syndrome; 22q11 microdeletion - CATCH 22; Syndrome/association with cardiac involvement; DiGeorge sequence; Friedreich's ataxia; Marfan syndrome; Noonan syndrome; Pompe's disease: glycogen storage disease type IIa; Tuberous sclerosis; Williams syndrome (infantile hypercalcaemia); Fetal rubella syndrome; Duchenne's muscular dystrophy; Muscular dystrophy; Ehlers-Danlos syndrome; Alagille syndrome: arteriohepatic dysplasia; Non-cardiac abnormality associated with heart disease; Non-cardiothoracic / vascular abnormality (DESCRIBE); Cystic fibrosis; Diaphragmatic hernia; Tracheo-oesophageal fistula; Omphalocoele; Duodenal stenosis/atresia; Sickle cell disease; Renal abnormality; Congenital coagulation disorder; Thoracic / mediastinal abnormality; Microcephaly; Choanal atresia; Tracheobronchial malacia; Hypothyroidism; Cerebral anomaly; Connective tissue disease; Kyphoscoliosis; Cleft lip / palate; Loeys-Dietz Syndrome (transforming growth factor beta receptor (TGFBR) gene); Von Willebrand disease; Maternally derived fetal disease or syndrome associated with heart disease; Major anomaly of gastrointestinal system; Multiple congenital malformations; Tracheal stenosis or disease
Severity of illness	Pre-procedural shock; Pre-procedural acidosis; Pre-procedural mechanical ventilatory or circulatory support; Preprocedural shock at time of surgery (persistent); Preprocedural cardiopulmonary resuscitation (< 48 hours); Cardiac Arrest; Respiratory failure;

ASD = atrial septal defect; AVSD = atrioventricular septal defect; DORV = double outlet right ventricle; HLHS = hypoplastic left heart syndrome; IVS = intact ventricular septum; PA = pulmonary artery; PAPVC = partial anomalous pulmonary venous connection; PDA = patent ductus arteriosus; TAPVC = total anomalous pulmonary venous connection; REV = reparation a letage ventriculaire; RV = right ventricle; TGA = transposition of the great arteries; UVH = univentricular heart; VSD = ventricular septal defect

BMJ Publishing Group Limited (BMJ) disclaims all liability and responsibility arising from any reliance placed on this supplemental material which has been supplied by the author(s)
