# Supplementary material for: External validation of the improving partial risk adjustment in surgery (PRAIS-2) model for 30-day mortality after paediatric cardiac surgery
Source: BMJ Open. 2020 Nov 27;10(11):e039236. doi: 10.1136/bmjopen-2020-039236 (PMC7703410; doi:10.1136/bmjopen-2020-039236)
Supplement: Supplementary data [file bmjopen-2020-039236supp006.pdf]

6a. Hosmer and Lemeshow goodness of fit groups, external validation cohort 2

PRAIS2 categories	Predicted survival	Predicted mortality	Observed survival	Observed mortality
[0.000131,0.00126]	119.93	0.07	120.00	0.00
(0.00126,0.00277]	119.76	0.24	120.00	0.00
(0.00277,0.00496]	118.54	0.46	119.00	0.00
(0.00496,0.0071]	119.28	0.72	120.00	0.00
(0.0071,0.0101]	118.97	1.03	119.00	1.00
(0.0101,0.0145]	117.59	1.41	118.00	1.00
(0.0145,0.0206]	117.94	2.06	120.00	0.00
(0.0206,0.0338]	115.78	3.22	114.00	5.00
(0.0338,0.0743]	114.04	5.96	117.00	3.00
(0.0743,0.408]	102.32	17.68	107.00	13.00

Supplementary Table 6b Hosmer and Lemeshow goodness of fit groups, external validation cohort 1

PRAIS2 categories	Predicted survival	Predicted mortality	Observed survival	Observed mortality
[3.61e-05,0.000938]	135.94	0.06	136.00	0.00
(0.000938,0.00249]	134.77	0.23	134.00	1.00
(0.00249,0.00482]	134.51	0.49	135.00	0.00
(0.00482,0.00803]	134.12	0.88	133.00	2.00
(0.00803,0.0115]	133.69	1.31	131.00	4.00
(0.0115,0.0163]	133.16	1.84	133.00	2.00
(0.0163,0.0221]	132.46	2.54	133.00	2.00
(0.0221,0.0305]	131.49	3.51	128.00	7.00
(0.0305,0.046]	130.01	4.99	127.00	8.00
(0.046,0.256]	124.00	12.00	130.00	6.00

6c. Hosmer and Lemeshow goodness of fit groups, overlapping cohorts

PRAIS2 categories	Predicted survival	Predicted mortality	Observed survival	Observed mortality
[4.29e-05,0.000909]	182.92	0.08	183.00	0.00
(0.000909,0.00234]	181.72	0.28	181.00	1.00
(0.00234,0.0045]	181.39	0.61	180.00	2.00
(0.0045,0.00754]	181.91	1.09	181.00	2.00
(0.00754,0.0108]	180.35	1.65	181.00	1.00
(0.0108,0.0159]	179.61	2.39	178.00	4.00
(0.0159,0.0229]	179.48	3.52	182.00	1.00
(0.0229,0.0327]	177.03	4.97	176.00	6.00
(0.0327,0.0539]	174.60	7.40	171.00	11.00
(0.0539,0.351]	164.34	18.66	155.00	28.00

6d. Hosmer and Lemeshow goodness of fit groups, in non-elective subset

PRAIS2 categories	Predicted survival	Predicted mortality	Observed survival	Observed mortality
[0.00109,0.00594]	48.82	0.18	49.00	0.00
(0.00594,0.00947]	47.64	0.36	48.00	0.00
(0.00947,0.0138]	47.46	0.54	47.00	1.00

(0.0138,0.0201]	47.20	0.80	48.00	0.00
(0.0201,0.0287]	47.80	1.20	47.00	2.00
(0.0287,0.0384]	46.44	1.56	44.00	4.00
(0.0384,0.0546]	45.83	2.17	46.00	2.00
(0.0546,0.0818]	44.71	3.29	47.00	1.00
(0.0818,0.126]	43.02	4.98	46.00	2.00
(0.126,0.408]	38.42	10.58	39.00	10.00

.
