# Supplementary material for: External validation of the improving partial risk adjustment in surgery (PRAIS-2) model for 30-day mortality after paediatric cardiac surgery
Source: BMJ Open. 2020 Nov 27;10(11):e039236. doi: 10.1136/bmjopen-2020-039236 (PMC7703410; doi:10.1136/bmjopen-2020-039236)
Supplement: Supplementary data [file bmjopen-2020-039236supp007.pdf]

**Supplementary table 7. Metrics of model discrimination according to different PRAIS-2 cut-off values in the Cohort 1**

| | Metrics | Top 90% | Top 80% | Top 20% | Top 10% |
|---|---|---|---|---|---|
| Cohort 1 | Cut off | 0.0009 | 0.0025 | 0.03 | 0.046 |
| | Sensitivity | 1 | 0.96 | 0.44 | 0.19 |
| | Specificity | 0.1 | 0.20 | 0.80 | 0.90 |
| | PPV | 0.03 | 0.03 | 0.05 | 0.04 |
| | NPV | 1 | 0.99 | 0.98 | 0.98 |
| | LRP | 1.11 | 1.21 | 2.23 | 1.89 |
| | LRN | 0 | 0.15 | 0.69 | 0.90 |

LRP= positive--likelihood ratios; LRN= negative-likelihood ratios; PPV= positive predictive values; NPV=negative predictive values

.
