# Supplementary material for: External validation of the improving partial risk adjustment in surgery (PRAIS-2) model for 30-day mortality after paediatric cardiac surgery
Source: BMJ Open. 2020 Nov 27;10(11):e039236. doi: 10.1136/bmjopen-2020-039236 (PMC7703410; doi:10.1136/bmjopen-2020-039236)
Supplement: Author's manuscript [file bmjopen-2020-039236.draft_revisions.pdf]

BMJ Open is committed to open peer review. As part of this commitment we make the peer review history of every article we publish publicly available.

When an article is published we post the peer reviewers' comments and the authors' responses online. We also post the versions of the paper that were used during peer review. These are the versions that the peer review comments apply to.

The versions of the paper that follow are the versions that were submitted during the peer review process. They are not the versions of record or the final published versions. They should not be cited or distributed as the published version of this manuscript.

BMJ Open is an open access journal and the full, final, typeset and author-corrected version of record of the manuscript is available on our site with no access controls, subscription charges or pay-per-view fees (http://bmjopen.bmj.com).

If you have any questions on BMJ Open's open peer review process please email info.bmjopen@bmj.com

BMJ Open

EXTERNAL VALIDATION OF THE IMPROVING PARTIAL RISK ADJUSTMENT IN SURGERY (PRAIS2) MODEL FOR 30-DAY MORTALITY AFTER PEDIATRIC CARDIAC SURGERY

Journal:	*BMJ Open*
Manuscript ID	bmjopen-2020-039236
Article Type:	Original research
Date Submitted by the Author:	13-Apr-2020
Complete List of Authors:	Cocomello, Lucia; University of Bristol, MRC Integrative Epidemiology Unit<br>Caputo, Massimo; University of Bristol, Bristol Heart Institute<br>Cornish, Rosie; University of Bristol, School of Social and Community Medicine<br>Lawlor, Debbie; Department of Social Medicine, University of Bristol, MRC Integrative Epidemiology Unit
Keywords:	Congenital heart disease < CARDIOLOGY, Cardiothoracic surgery < SURGERY, Paediatric cardiology < CARDIOLOGY, Paediatric cardiac surgery < PAEDIATRIC SURGERY, Paediatric cardiothoracic surgery < PAEDIATRIC SURGERY

SCHOLARONE™
Manuscripts

I, the Submitting Author has the right to grant and does grant on behalf of all authors of the Work (as defined in the below author licence), an exclusive licence and/or a non-exclusive licence for contributions from authors who are: i) UK Crown employees; ii) where BMJ has agreed a CC-BY licence shall apply, and/or iii) in accordance with the terms applicable for US Federal Government officers or employees acting as part of their official duties; on a worldwide, perpetual, irrevocable, royalty-free basis to BMJ Publishing Group Ltd ("BMJ") its licensees and where the relevant Journal is co-owned by BMJ to the co-owners of the Journal, to publish the Work in this journal and any other BMJ products and to exploit all rights, as set out in our licence.

The Submitting Author accepts and understands that any supply made under these terms is made by BMJ to the Submitting Author unless you are acting as an employee on behalf of your employer or a postgraduate student of an affiliated institution which is paying any applicable article publishing charge ("APC") for Open Access articles. Where the Submitting Author wishes to make the Work available on an Open Access basis (and intends to pay the relevant APC), the terms of reuse of such Open Access shall be governed by a Creative Commons licence – details of these licences and which Creative Commons licence will apply to this Work are set out in our licence referred to above.

Other than as permitted in any relevant BMJ Author's Self Archiving Policies, I confirm this Work has not been accepted for publication elsewhere, is not being considered for publication elsewhere and does not duplicate material already published. I confirm all authors consent to publication of this Work and authorise the granting of this licence.

For peer review only

EXTERNAL VALIDATION OF THE IMPROVING PARTIAL RISK ADJUSTMENT IN SURGERY (PRAIS2) MODEL FOR 30-DAY MORTALITY AFTER PEDIATRIC CARDIAC SURGERY

Corresponding Author:

Lucia Cocomello,

MRC Integrative Epidemiology Unit,

Oakfield House, Oakfield Grove, Bristol, BS8 2BN, United Kingdom

nn18747@bristol.ac.uk;

Massimo Caputo

Bristol Heart Institute,

Terrell St, Bristol, BS2 8 ED, United Kingdom
M.Caputo@bristol.ac.uk;

Rosie Cornish
MRC Integrative Epidemiology Unit,

Oakfield House, Oakfield Grove, Bristol, BS8 2BN, United Kingdom;

Population Health Sciences, Bristol Medical School, University of Bristol, United Kingdom.

rosie.cornish@bristol.ac.uk

Deborah A. Lawlor

MRC Integrative Epidemiology Unit,

Oakfield House, Oakfield Grove, Bristol, BS8 2BN, United Kingdom;

Population Health Sciences, Bristol Medical School, University of Bristol, United Kingdom;

Bristol NIHR Biomedical Research Centre.

d.a.lawlor@bristol.ac.uk

KEYWORDS: congenital heart disease, paediatric cardiac surgery, risk prediction

Word count:2780

For peer review only

ABSTRACT

Objective: Risk stratification in paediatric patients undergoing heart surgery remains a challenge. The improving partial risk adjustment in surgery (PRAIS2) is a risk model predicting 30-day mortality which has been recently developed and validated using a UK-based cohort from April 2009-March 2015. We aimed to perform an independent temporal external validation to explore its generalisability and clinical utility.

Methods: PRAIS2 validation was carried out using a single centre (Bristol, UK) cohort from April 2004 to March 2009 and April 2015 to July 2019. For each subject PRAIS2 score was calculated according to the original formula. PRAIS2 performance was assessed in terms of discrimination by means of ROC curve analysis and calibration by using the calibration belt method.

Results: A total of 1330 (2004-2009) and 1187 (2015-2019) paediatric cardiac surgical procedures were included in the first and second independent validation, respectively (median age at the procedure 6.0 and 6.9 months). PRAIS2 score showed excellent discrimination for both independent validations (AUC 0.72 (95%CI: 0.65 to 0.80) and 0.87 (95%CI: 0.82 to 0.93), respectively). While PRAIS2 was only marginally calibrated in the first validation, with a tendency to underestimate risk P-value = 0.051), the second validation showed good calibration with 95% confidence belt containing the bisector for predicted mortality (P-value = 0.15); We also observed good performance in the subgroup of patients undergoing non-elective procedures (N = 482; AUC 0.78 (95%CI 0.68 to 0.87); Calibration belt containing the bisector (P-value=0.61).

Conclusions: In a single centre UK-based cohort, PRAIS2 showed excellent discrimination and calibration in predicting 30-day mortality in paediatric cardiac surgery including in those undergoing non-elective procedures. Our results support a wider adoption of PRAIS2 score in the clinical practice.

For peer review only

Strengths and limitations of this study

- A strength of the present study is that data were prospectively collected as part of the UK National Congenital Heart Disease Audit and as such they undergo continuous and inclusive systematic validation that includes the review of a sample of case notes by external auditors to ensure coding accuracy.
- We used a recently proposed method (calibration belt) which does not require patients to be categorised according to risk percentile but rather provides a risk function across all risk value with relative uncertainty measure (95% CI)
- A key limitation of this study is that the sample size is relatively small and considerably smaller than the cohort used to develop PRAIS2

Key questions

- **What is already known about this subject?** The improving partial risk adjustment in surgery (PRAIS2) is a risk model predicting 30-day mortality which has been recently developed and validated using a UK-wide cohort.
- **What does this study add?** The present study reported the first independent external validation of the PRAIS2 using a single centre cohort which confirmed excellent performance of the model and for the first time showed that it also accurately predicts mortality in patients undergoing non-elective procedures
- **How might this impact on clinical practice?** Our results support a wider adoption of the PRAIS2 in the clinical practice.

INTRODUCTION

Congenital heart diseases (CHD) are the most common birth defect affecting between 6-8 per 1000 of live born children in middle and high income countries.[1,2] Around 5000 paediatric cardiac surgical procedures are performed each year in the United Kingdom. Despite overall mortality being low (3%), there is notable variation in mortality rates between

For peer review only

centres. Mortality rates, and variation between centres, are monitored by public and healthcare regulatory bodies as part of assessing the quality of care provided by the centres. In order to monitor a centre's quality of care and performance and make fair comparisons between centres in terms of mortality following paediatric cardiac surgery it is essential to take account of differences in case mix across centres.[3] This requires accurate risk stratification. However, CHD includes a large spectrum of diagnoses with a wide range of surgical procedures performed in the context of a relatively small number of patients. These characteristics make risk stratification extremely challenging.

Several methods that aim to provide an objective risk assessment for taking account of case mix when assessing centre performance have been developed. These include consensus based methods, such as the risk adjusted classification for congenital heart surgery (RACHS-1) [4] and Aristotle,[5] and more recently empirical research based methods, such as the society of thoracic surgeons-European association of cardiothoracic surgery score (STS-EACTS)[6] and the partial risk adjustment in surgery (PRAIS)[7], which has been developed in the UK and proposed for measuring between centre variation in mortality across the UK. The most recently updated version, PRAIS2[8], was developed to predict 30-day mortality using data from the UK National Congenital Heart Disease Audit (NCHDA).[9] In comparison to PRAIS, PRAIS2 included more detailed information about acuity, diagnosis and comorbidities and was shown to have better discrimination and calibration than the original PRAIS[10]. However, adoption of PRAIS2 into clinical practice is still limited and this has been partially attributed to the lack of external independent validation other than the one presented by the authors in the original paper.[11]That validation used data from the same UK dataset but was undertaken on a temporally independent sub-cohort (Figure 1). Whilst such validation within a study is important, using the same cohort (and same group of investigators) can result in risk of optimism bias, which would tend to exaggerate risk stratification accuracy in comparison to findings from independent cohorts and investigators.[12],[13] Moreover, prediction models can present significant calibration drift due to

For peer review only

temporal and geographic differences in case mix, patients characteristics and surgical technique; the impact of this on the performance of PRAIS2 is unknown.[14 15] Lastly, as the data used to develop PRAIS2 did not include information on whether the procedures were elective or conducted as non-elective (emergencies, urgency and salvage) it was not possible to determine whether performance was similar in both of these situations. It is not implausible that stratification accuracy will differ between the two.

The purpose of this study was to perform an external independent validation of the PRAIS2 score in a cohort from a single tertiary paediatric UK centre (in Bristol, South West England). This single UK centre contributed to the cohort in which PRAIS2 was originally developed but with data from different time periods than used here, where we explore performance separately in two cohorts: (i) undergoing procedures earlier than those used in PRAIS2 and (ii) undergoing procedures after those used in PRAIS2. To explore possible calibration drift, we have compared calibration of the model prediction between the three Bristol Heart Institute cohorts; procedures undertaken 2004-2009, 2009-2015 (included in PRAIS2 development) and 2015 to 2019. In a subsample, we were also able to undertake the first (exploratory) analysis of how well PRAIS2 performs in those undergoing non-elective procedures.

METHODS

The study complies with the Declaration of Helsinki. This study was approved by the institutional review board (IRB) at the Bristol Heart Institute (reference number 250868). As this analysis came under clinical audit / quality of care assessment and all data were anonymised following the governance criteria of the NHS, the IRB agreed informed consent was not required.

Patient and public involvement

This research was done without Patient and Public Involvement.

Data Source

For peer review only

Data were obtained from the Bristol dataset which is part of the National Congenital Heart Disease Audit (NCHDA) within the National Institute of Cardiovascular Outcomes Research (NICOR). As such they undergo continuous and inclusive systematic validation of the data used here, which includes the review of a sample of case notes by external auditors to ensure coding accuracy.[9] **Figure 1** summarises the data sources used in this study and their relationship to the UK National data used in the development of PRAIS2. The Bristol cohort includes data from patients undergoing procedures between April 2004 and July 2019. PRAIS2 was developed using national data (including that from the Bristol cohort) from patients undergoing procedures between April 2009 and March 2015. In these analyses we use Bristol data from April 2004 to March 2009 (N = 1330) and April 2015 to July 2019 (N = 1187) as independent external validation (hereafter referred to as independent validation 1 and 2, respectively). Whilst we acknowledge that treatments and mortality rates have changed since 2004, and the first validation set may not reflect contemporary practice, we would expect this to primarily affect model calibration; finding good discrimination for this earlier set would support external validity and generalisability of PRAIS-2. Seeing good discrimination across all time periods would provide valuable evidence about the generalisability of the PRAIS-2 model. In addition, we explored discrimination and calibration in the Bristol cohort that was also included in the national cohort to develop PRAIS2, April 2009 to March 2015 (N = 1794). This allows us to explore any evidence of geographical (centre) difference in stratification performance and using all three cohorts to explore the extent of temporal calibration drift.

The Bristol dataset included 4,885 paediatric cardiac surgical procedures (defined as surgery on the heart or great vessel in patients aged < 16 years old, excluding catheter procedures and trivial/minor procedures). Of these 4,885 we had to exclude 575 (12%) for missing data; the remaining 4310 contributed to one of the three temporal cohorts used here.

PRAIS2 prediction score and its constituent variables

PRAIS2 score is generated from a transformed logistic regression model of 30-day mortality following cardiac surgery.[10] The model included the following perioperative variables: age,

For peer review only - http://bmjopen.bmj.com/site/about/guidelines.xhtml

weight, diagnosis, procedure group, type of procedure, whether or not there was definite univentricular heart function, additional cardiac risk factors, acquired comorbidity, congenital comorbidity, severity of illness and an additional coefficient for procedures performed after 2013 (Supplementary Table 1 shows the units or categories of each of these variables). The formula for the PRAIS2 score (using the function from the logistic regression model) is

$$\frac{1}{1+e^{-z}}$$

Where z is the logistic model function of the nine variables. In all analyses we used the PRAIS2 model (equation) that was recalibrated by the original authors after their internal validation (see Supplementary Equation 1).

Outcome

The primary outcome was 30-day all-cause mortality. Information on mortality was obtained from the Office for National Statistics (ONS).

Statistical analysis

Continuous data were presented as medians with interquartile ranges (IQR). Categorical variables were presented as counts with percentages. We assessed the discriminative ability of the PRIAS2 model to distinguish between children who did and did not die within 30-days using the area under the receiver operating characteristic curve (AUC) analysis[16] and its calibration by comparing observed to predicted 30-day mortality rates for groups of patients with different predicted levels of risk.[11 17 18] Overall model calibration was assessed using the calibration belt method. The method is based on a generalisation of Cox's regression modelling where the relation between the logits of the probability predicted by a model and of the event rates observed in a sample is represented by a polynomial function whose coefficients are fitted and its degree is fixed by a series of likelihood-ratio tests.[19] Risk underestimation is suggested if the calibration belt with its 95% confidence interval (CI) is

For peer review only

above the bisector (perfect prediction line) while overestimation is indicated by the belt with its 95% CI being below the bisector. The plot was accompanied by the Hosmer-Lemeshow test goodness of fit test statistical test.[20] All analyses were performed using R version 3.5.2.

RESULTS

Our analyses included 1125 patients undergoing 1330 procedures and 902 patients undergoing 1187 procedures in the external validation samples 1 and 2, respectively, and a subset (from validation 2) of 281, undergoing 482 non-elective procedures. Median age at the time of the first procedure of any participant included in any analyses was 6.5 months (IQR 1.6 – 42.5).

Table 1 shows patient characteristics of the two external validation cohorts and the subgroup undergoing non-elective procedures together with the Bristol cohort that was included in the national cohort used to develop PRAIS2.

There was a trend towards an increased risk profile (increasing in the number of complex procedures, group 1) in the most recent cohort and a subsequent increase in the expected mortality (PRAIS2). Notably, the observed mortality trend was in the opposite direction, with a reduction in the most recent cohort.

For peer review only

Table 1. Patient characteristics in the earlier, external validation cohorts and in the non-elective subset. Definitions of variables are given in Supplementary Table 1

	Independent validation 1 (April 2004 - March2009)	Independent validation 2 (April 2015 - July 2019)	Non-elective subset (April 2015 - July 2019)	Cohort overlapping with PRAIS2 cohort (April 2009- March 2015)
**Total**	(n=1330 procedures n=1125 patients)	(n=1187 procedures n=902 patients)	(n=482 procedures n=281 patients)	(n=1794 procedures N=1461 patients)
**Age at procedure median, months [IQR]**	6.0 [1.4 - 27.7]	6.9 [2.2-49.9]	4.8 [1.1-35.5]	6.0 [1.4-36.4]
**Weight median, Kg [IQR]**	6.0 [3.4 – 12.0]	6.9 [3.9-15.3]	3.7 [3.1-5.3]	6 [3.6-12.6]
**Diagnoses group n (%)[a]**				
GROUP1	69 (5.1)	107 (9.0)	47 (9.8)	153 (8.5)
GROUP2	149 (11.2)	161 (13.6)	77 (16.0)	192 (10.7)
GROUP 3	109 (8.2)	103 (8.7)	65 (13.5)	124 (9.3)
GROUP 4	215 (16.2)	232 (19.5)	118 (24.5)	222 (12.4)
GROUP 5	104 (7.8)	122 (10.3)	49 (10.2)	119 (6.6)
GROUP 6	98 (7.4)	131 (11.0)	44 (9.1)	89 (4.9)
GROUP 7	175 (13.1)	127 (10.7)	34 (7.1)	309 (17.2)
GROUP 8	150 (11.3)	103 (8.7)	32 (6.6)	186 (10.4)
GROUP 9	20 (1.5)	<5 (<1)	<5 (<1)	25 (1.4)
GROUP 10	56 (4.2)	14 (1.2)	6 (1.2)	94 (5.2)
GROUP 11	185 (13.9)	85 (7.2)	8 (1.7)	281 (15.6)

For peer review only

Procedure Group n (%)[a]				
GROUP 1	<5 (<1)	31 (2.6)	22 (4.6)	27 (1.5)
GROUP 2	29 (2.2)	11 (0.9)	10 (2.1)	33 (1.8)
GROUP 3	105 (7.9	46 (3.9)	34 (7.1)	84 (4.6)
GROUP 4	99 (7.4)	103 (8.7)	73 (15.1)	114 (6.3)
GROUP 5	236 (17.8)	228 (19.2)	126 (26.1)	344 (19.1)
GROUP 6	148 (11.1)	53 (4.5)	36 (7.5)	179 (9.9)
GROUP 7	21 (1.6)	44 (3.7)	16 (3.3)	49 (2.7)
GROUP 8	79 (5.9)	96 (8.1)	12 (2.5)	103 (5.7)
GROUP 9	20 (1.5)	16 (1.3)	5 (1.0)	26 (1.4)
GROUP 10	36 (2.7)	35 (2.9)	7 (1.5)	70 (3.9)
GROUP 11	36 (2.7)	19 (1.6)	8 (1.7)	57 (3.1)
GROUP 12	32 (2.4)	63 (5.3)	<5 (<1)[b]	51 (2.8)
GROUP 13	80 (6.0)	66 (5.6)	5 (1.0)	103 (5.7)
GROUP 14	38 (2.8)	43 (3.6)	7 (1.5)	55 (3.0)
GROUP 15	216 (16.2)	144 (12.1)	11 (2.3)	273 (13.8)
GROUP 20	154 (11.6)	189 (15.9)	109 (22.6)	226 (12.5)
**Severity of illness n(%)**	0 (0)	221 (18.6)	202 (41.9)	86 (4.7)
**Acquired comorbidity n (%)**	9 (0.7)	205 (17.3)	131 (27.2)	94 (5.2)
**Congenital comorbidity n (%)**	172 (12.9)	341 (28.7)	143 (29.7)	268 (14.9)
**Additional cardiac risk factors n (%)**	58 (4.4)	81 (6.8)	55 (11.4)	52 (2.8)
**UVH category n (%)**	141 (10.6)	217 (18.3)	95 (19.7)	258 (14.3)

For peer review only - http://bmjopen.bmj.com/site/about/guidelines.xhtml

**Bypass n (%)**	884 (66.4)	902 (76.0)	272 (56.4)	1333 (74.3)
**PRAIS2 mean [SD]**	2.1% [±2.8]	2.7% [±4.0]	5.2% [±4.2]	2.2% [±3.3]
**30-day mortality**	31 (2.3)	23 (1.9)	22 (4.6)	56 (3.1)

[a] as the diagnoses groups and procedure groups have lengthy text details, we have not provided them in the table; they can be found in Supplementary Table 1.
[b] Exact number suppressed for disclosure control purposes

For peer review only

The predicted risk in the external validation cohorts 1 and 2, the PRAIS2 overlapping cohort and the non-elective subset were 2.1%,2.7%, 5.2% and 2.2% respectively and the observed mortality was 2.3%, 1.9%, 4.6% and 3.1%) respectively. Individual risk distribution among those who died and those who survived is shown in Supplementary Figure 1.

In the cohort 2 external validation analysis, the PRAIS2 score showed good discrimination ability (AUC 0.87; 95%CI 0.82-0.93, Figure 2b) and calibration between predicted and observed mortality across all risk categories with 95% confidence interval of calibration belt containing the bisector (p value=0.16,; Figure 3b;. Hosmer and Lemeshow goodness of fit test chi-squared=7.60, df=8, P-value 0.47, Supplementary Table 2b).

In cohort 1, PRAIS2 showed a lower but still acceptable discrimination ability (AUC 0.72 (95%CI: 0.65 to 0.80, Figure 2a) and was only marginally calibrated according to the calibration belt method (p-value=0.051, Figure 3a; Hosmer and Lemeshow goodness of fit test chi-squared=15.80, df=8, P-value 0.045, Supplementary Table 2a).

In additional analyses to check that data from Bristol were not notably different to the main UK wide discovery cohort, we showed that the cohort from Bristol that contributed to the original PRAIS2 discovery also had good discrimination, with results similar to the whole PRAIS2 discovery cohort (AUC 0.82 (95%CI 0.77-0.88, Figure 2c). However, the model was only marginally calibrated with a tendency to risk underestimation (Figure 3c). Hosmer and Lemeshow goodness of fit test chi-squared =21.90; df=8; P-value=0.0051; Supplementary Table 2c).

In the subgroup of patients undergoing non-elective procedures, the model showed good discrimination, with AUC 0.78 (95%CI 0.68-0.87, Figure 4a) and good calibration, with the bisector contained within the 95% confidence level of the calibration belt. (p-value 0.61; Figure 4b and Hosmer and Lemeshow goodness of fit test, chi-squared=10.04, df=8, P-value=0.26; Supplementary Table 2d). We were not able to conduct the analysis restricted to elective procedures as only one participant died in this subset.

For peer review only

DISCUSSION

Risk prediction models play an important role in current paediatric cardiac surgical practice, because they allow meaningful comparison of outcomes between institutions by adjusting for differing case-mix. Moreover, they can also give useful information for surgical decision making, preoperative patient information and quality assurance measures. Before implementing a risk model in clinical practice, it is important to confirm its prediction ability with external validation.[11 21]

To our knowledge PRAIS2 has never undergone external validation other than that originally presented by the authors who developed the model. It is important that prediction models are validated by independent researchers, as well as in independent samples, before being adopted into clinical practice because authors evaluating the performance of their own model may tend to be overly optimistic in interpreting results or selective reporting.[13 22] For this reason we conducted an external validation study of PRAIS2 in two cohorts from the paediatric cardiac surgical procedures cohort at the Bristol Royal Hospital for Children. Our findings show that in these two, independent (of the original cohort used to develop PRAIS2), cohorts of 1330 procedures (in 1125 patients) and 1187 procedures (in 902 patients), respectively, the PRAIS2 model has good discrimination. Overall, the model showed good calibration. Remarkably, the model showed the best calibration in the most recent cohort. These findings do not suggest a relevant calibration drift. However, a further validation of the PRAIS2 in the coming years is recommended as calibration drift is plausible and can result in risk overestimation.[15]

As PRAIS2 was developed with data from across the UK and in our external validation we have only used data from Bristol we wanted to make sure that prediction in Bristol was broadly consistent with the UK as a whole. We therefore explored the predictive accuracy of PRAIS2 in the Bristol cohort that contributed to the original PRAIS2 discovery and found good discrimination, but the model was only marginally calibrated with a tendency to underestimate risk. It is not uncommon for calibration to differ between cohorts from different regions or over time. We are not able

For peer review only

to determine variation in calibration between all of the different centres that contributed to the original PRAIS2 development as data were not presented by centre.[10] It is interesting that a more recent Bristol cohort was better calibrated to PRAIS2 than the original Bristol cohort that contributed to PRAIS2 development. This could be partially explained by improved surgical results during recent years in our centre.

PRAIS2 development excluded non-elective procedures and so its accuracy in this group, or whether this would be a valuable predictor, is unknown. We demonstrated that PRAIS2 does have good discrimination and calibration in this higher risk subgroup. However, we acknowledge that our numbers were small for these analyses and this needs further exploration and validation in larger independent samples. We were not able to compare performance between those undergoing elective procedures and those undergoing non-elective procedures as there were insufficient deaths in those undergoing elective procedures. This marked difference in mortality between elective and non-elective procedures suggests that elective vs non-elective status might be a valuable covariable in PRAIS2, but large cohorts with these data would be needed to determine this.

A strength of the present study is that data were prospectively collected as part of the NCHDA national audit and as such they undergo continuous and inclusive systematic validation that includes the review of a sample of case notes by external auditors to ensure coding accuracy.[9] A key limitation of this study is that the sample size is relatively small and considerably smaller than the cohort used to develop PRAIS2, which included a total of 21838 procedures (combining discovery and validation). Unlike the development cohort that included all UK patients, we only have data from one centre located in the South West of England where the population are more affluent and less ethnically mixed in comparison to the UK as a whole. Hence further replication in a larger and more diverse population is necessary.

For peer review only

The Hosmer and Lemeshow goodness of fit test for calibration is widely used in assessing calibration,[23 24] but it provides a statistical test result comparing predicted to observed outcome rates across arbitrary grouping of deciles of risk, which may have limited clinical applicability. Therefore, we used a recently proposed method (calibration belt) which does not require patients to be categorised but rather compares across the continuous score of risk for our main method of assessing calibration.[25] We also presented Hosmer and Lemeshow goodness of fit test in the supplementary material for comparison with any previous studies.

In conclusion, our study shows good external validity of PRAIS2 for predicting short-term mortality in paediatric cardiac surgery (the outcome it was developed to predict). We also show preliminary evidence that PRAIS2 accurately predicts short-term mortality in non-elective procedures. If similar results were found in more diverse external populations, including those outside of the UK, its use as the standard tool for risk prediction in paediatric patients undergoing cardiac surgery would be recommended.

Acknowledgments

We thank Mai Baquedano for support with data management

Funding

This study was supported by the British Heart Foundation Accelerator Award (AA/18/7/34219) and the Bristol National Institute of Health Research Biomedical Research Centre. LC, RC, and DAL work in a unit that receives support from the University of Bristol and the UK Medical Research Council (MC_UU_00011/6). DAL is a National Institute of Health Research Senior Investigator (NF-0616-10102). The views expressed in this

For peer review only

publication are those of the author(s) and not necessarily those of the UK National Health Service, the National Institute for Health Research or the UK Department of Health and Social Care, or any other funders mentioned here.

Conflict

DAL has received support from several national and international charity and government grants and from Medtronic Ltd and Roche Diagnostics for research unrelated to that presented here. The other authors declare no conflicts of interest.

For peer review only

Figure legend

Figure 1. Patients included in the study

Figure2. ROC curve for PRAIS2 in the independent validation 1 and 2 (2a and 2b) and overlapping (2c) cohorts

Figure3a. Calibration belt of PRAIS2 in the independent validation 1

Figure3b. Calibration belt of PRAIS2 in the independent validation 2

Figure3c. Calibration belt of PRAIS2 in the and overlapping cohorts

Figure 4a. AUC in the subset of non-elective procedures in the external validation cohort.

Figure 4b. Calibration belt in the subset of non-elective procedures in the external validation cohort.

Supplementary Figure 1. Sinaplot of PRAIS2 distribution stratified for incidence of 30-day mortality.

For peer review only

REFERENCES

1. Hoffman JI, Kaplan S. The incidence of congenital heart disease. *J Am Coll Cardiol* 2002;39(12):1890-900. doi: 10.1016/s0735-1097(02)01886-7 [published Online First: 2002/06/27]
2. van der Linde D, Konings EE, Slager MA, et al. Birth prevalence of congenital heart disease worldwide: a systematic review and meta-analysis. *J Am Coll Cardiol* 2011;58(21):2241-7. doi: 10.1016/j.jacc.2011.08.025 [published Online First: 2011/11/15]
3. Brown KL, Crowe S, Franklin R, et al. Trends in 30-day mortality rate and case mix for paediatric cardiac surgery in the UK between 2000 and 2010. *Open Heart* 2015;2(1) doi: UNSP e000157

10.1136/openhrt-2014-000157
4. Jenkins KJ, Gauvreau K, Newburger JW, et al. Consensus-based method for risk adjustment for surgery for congenital heart disease. *J Thorac Cardiovasc Surg* 2002;123(1):110-8. doi: 10.1067/mtc.2002.119064 [published Online First: 2002/01/10]
5. Lacour-Gayet F, Clarke D, Jacobs J, et al. The Aristotle score for congenital heart surgery. *Semin Thorac Cardiovasc Surg Pediatr Card Surg Annu* 2004;7:185-91. doi: 10.1053/j.pcsu.2004.02.011 [published Online First: 2004/07/31]
6. O'Brien SM, Clarke DR, Jacobs JP, et al. An empirically based tool for analyzing mortality associated with congenital heart surgery. *J Thorac Cardiovasc Surg* 2009;138(5):1139-53. doi: 10.1016/j.jtcvs.2009.03.071 [published Online First: 2009/10/20]
7. Pagel C, Brown KL, Crowe S, et al. A mortality risk model to adjust for case mix in UK paediatric cardiac surgery. Southampton (UK)2013.
8. Rogers L, Brown KL, Franklin RC, et al. Improving Risk Adjustment for Mortality After Pediatric Cardiac Surgery: The UK PRAiS2 Model. *Ann Thorac Surg* 2017;104(1):211-19. doi: 10.1016/j.athoracsur.2016.12.014 [published Online First: 2017/03/21]
9. Wang T, Chen L, Yang T, et al. Congenital Heart Disease and Risk of Cardiovascular Disease: A Meta-Analysis of Cohort Studies. *J Am Heart Assoc* 2019;8(10):e012030. doi: 10.1161/JAHA.119.012030 [published Online First: 2019/05/10]
10. Pagel C, Rogers L, Brown K, et al. Improving risk adjustment in the PRAiS (Partial Risk Adjustment in Surgery) model for mortality after paediatric cardiac surgery and improving public understanding of its use in monitoring outcomes. Southampton (UK)2017.
11. Altman DG, Vergouwe Y, Royston P, et al. Prognosis and prognostic research: validating a prognostic model. *BMJ* 2009;338:b605. doi: 10.1136/bmj.b605 [published Online First: 2009/05/30]
12. Siontis GC, Tzoulaki I, Siontis KC, et al. Comparisons of established risk prediction models for cardiovascular disease: systematic review. *BMJ* 2012;344:e3318. doi: 10.1136/bmj.e3318 [published Online First: 2012/05/26]
13. Ban JW, Stevens R, Perera R. Predictors for independent external validation of cardiovascular risk clinical prediction rules: Cox proportional hazards regression analyses. *Diagn Progn Res* 2018;2:3. doi: 10.1186/s41512-018-0025-6 [published Online First: 2018/02/06]

For peer review only

14. Hickey GL, Blackstone EH. External model validation of binary clinical risk prediction models in cardiovascular and thoracic surgery. *J Thorac Cardiovasc Surg* 2016;152(2):351-5. doi: 10.1016/j.jtcvs.2016.04.023 [published Online First: 2016/05/25]
15. Hickey GL, Grant SW, Murphy GJ, et al. Dynamic trends in cardiac surgery: why the logistic EuroSCORE is no longer suitable for contemporary cardiac surgery and implications for future risk models. *Eur J Cardio-Thorac* 2013;43(6):1146-52. doi: 10.1093/ejcts/ezs584
16. Hanley JA, Mcneil BJ. The Meaning and Use of the Area under a Receiver Operating Characteristic (Roc) Curve. *Radiology* 1982;143(1):29-36. doi: DOI 10.1148/radiology.143.1.7063747
17. Harrell FE, Lee KL, Mark DB. Multivariable prognostic models: Issues in developing models, evaluating assumptions and adequacy, and measuring and reducing errors. *Stat Med* 1996;15(4):361-87. doi: Doi 10.1002/(Sici)1097-0258(19960229)15:4<361::Aid-Sim168>3.0.Co;2-4
18. Mackillop WJ, Quirt CF. Measuring the accuracy of prognostic judgments in oncology. *J Clin Epidemiol* 1997;50(1):21-29. doi: Doi 10.1016/S0895-4356(96)00316-2
19. Nattino G, Finazzi S, Bertolini G. A new calibration test and a reappraisal of the calibration belt for the assessment of prediction models based on dichotomous outcomes. *Stat Med* 2014;33(14):2390-407. doi: 10.1002/sim.6100
20. Fagerland MW, Hosmer DW. A goodness-of-fit test for the proportional odds regression model. *Stat Med* 2013;32(13):2235-49. doi: 10.1002/sim.5645 [published Online First: 2012/10/06]
21. Bleeker SE, Moll HA, Steyerberg EW, et al. External validation is necessary in prediction research: a clinical example. *J Clin Epidemiol* 2003;56(9):826-32. doi: 10.1016/s0895-4356(03)00207-5 [published Online First: 2003/09/25]
22. Collins GS, de Groot JA, Dutton S, et al. External validation of multivariable prediction models: a systematic review of methodological conduct and reporting. *Bmc Med Res Methodol* 2014;14 doi: Artn 40

10.1186/1471-2288-14-40
23. Cook NR. Statistical evaluation of prognostic versus diagnostic models: Beyond the ROC curve. *Clin Chem* 2008;54(1):17-23. doi: 10.1373/clinchem.2007.096529
24. Nashef SAM, Rogues F, Michel P, et al. European system for cardiac operative risk evaluation (EuroSCORE). *Eur J Cardio-Thorac* 1999;16(1):9-13. doi: Doi 10.1016/S1010-7940(99)00134-7
25. Steyerberg EW, Vickers AJ, Cook NR, et al. Assessing the Performance of Prediction Models A Framework for Traditional and Novel Measures. *Epidemiology* 2010;21(1):128-38. doi: 10.1097/EDE.0b013e3181c30fb2

Contributorship Statement:

Lucia Cocomello: conception and design, data acquisition, analysis and interpretation of data, writing publication

Massimo Caputo: Critical revision of publication, approval of final publication

Rosie Cornish: Critical revision of publication, approval of final publication

For peer review only

Deborah Lawlor: Writing Publication, Critical revision of publication, approval of final publication, supervision

For peer review only

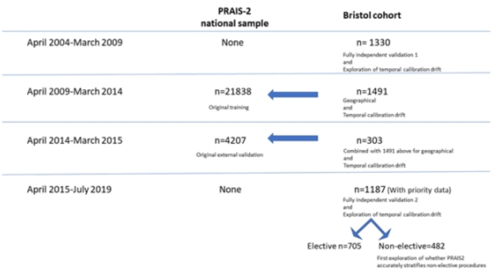

Figure 1. Patients included in the study

108x60mm (300 x 300 DPI)

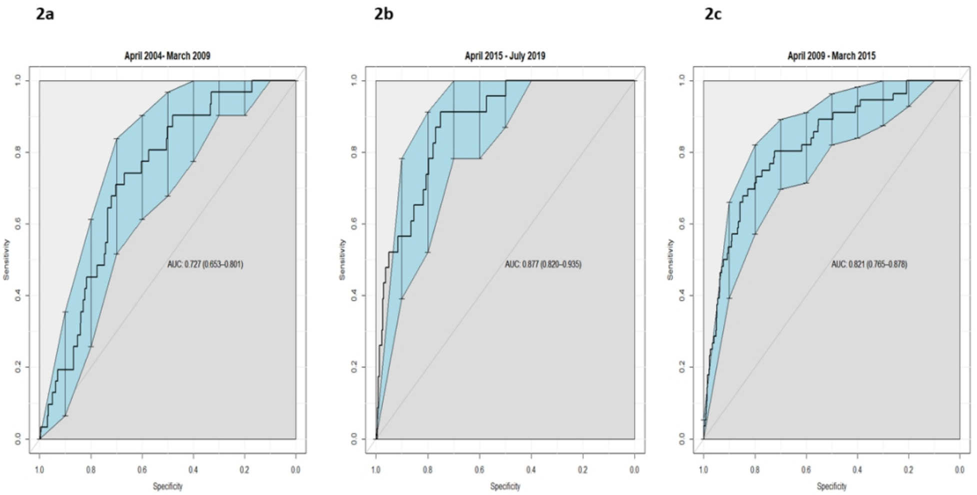

Figure2. ROC curve for PRAIS2 in the independent validation 1 and 2 (2a and 2b) and overlapping (2c) cohorts

108x60mm (300 x 300 DPI)

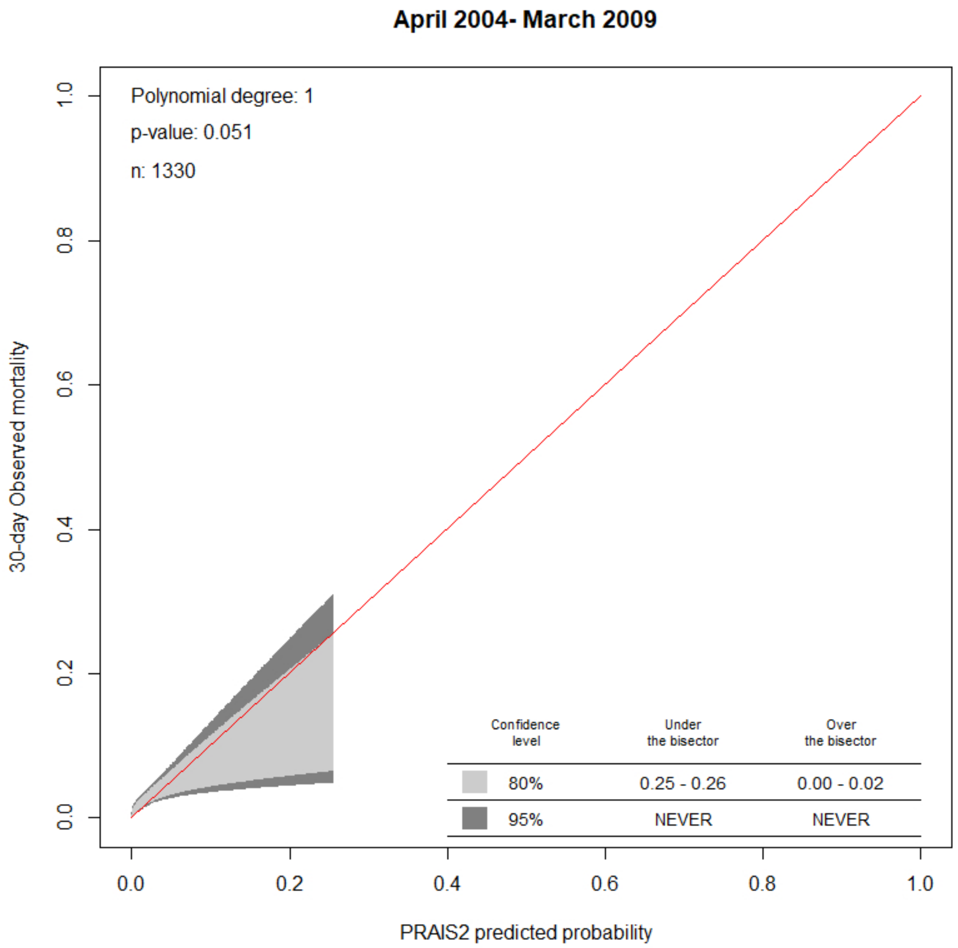

Figure3a. Calibration belt of PRAIS2 in the independent validation 1

67x67mm (300 x 300 DPI)

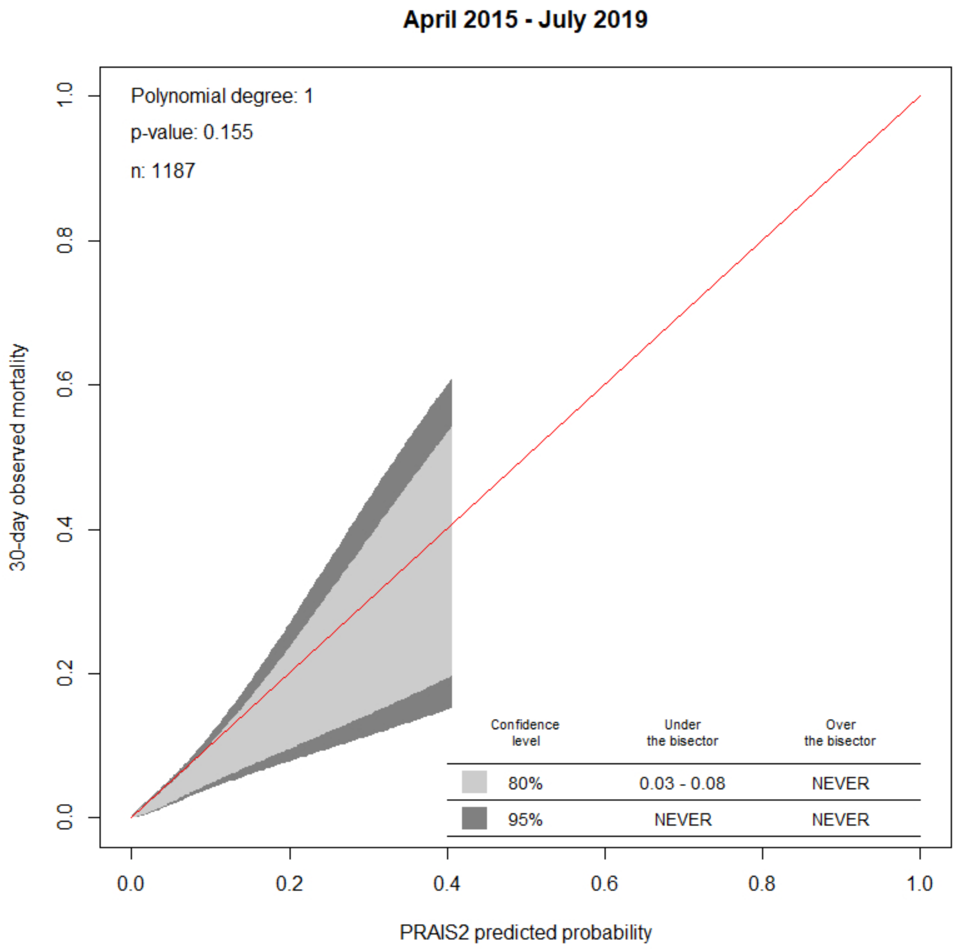

Figure3b. Calibration belt of PRAIS2 in the independent validation 2

67x67mm (300 x 300 DPI)

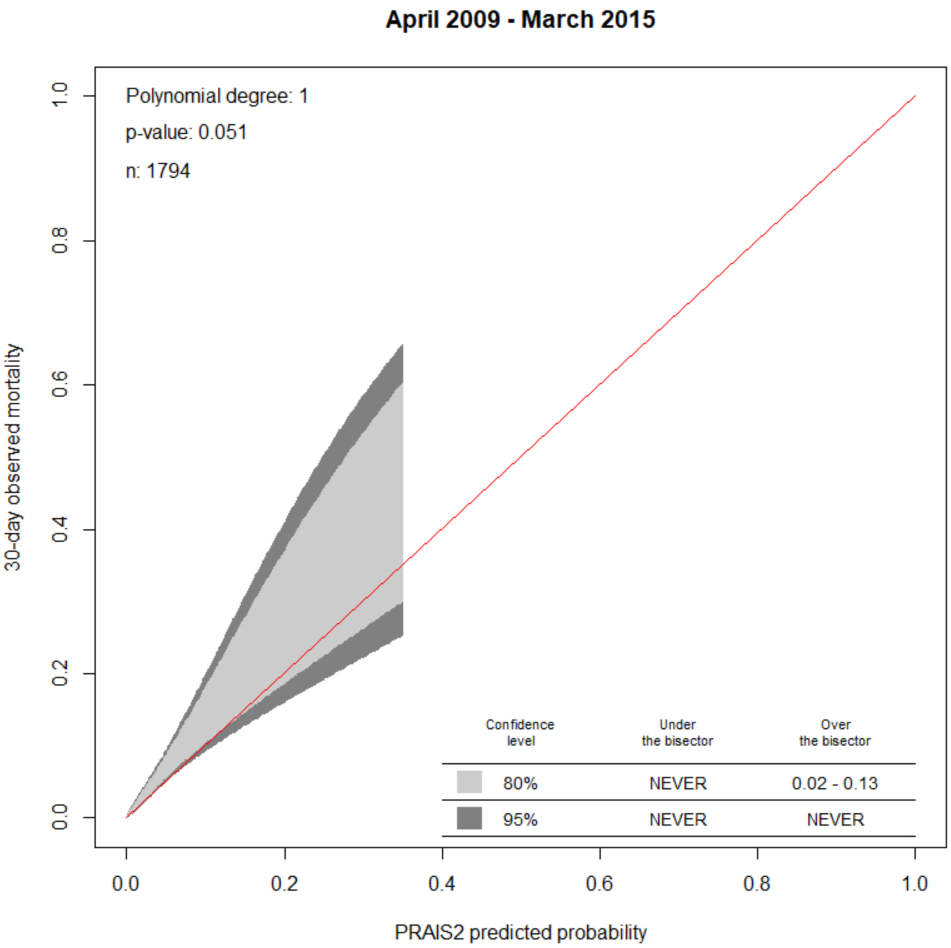

Figure3c. Calibration belt of PRAIS2 in the overlapping cohorts

67x67mm (300 x 300 DPI)

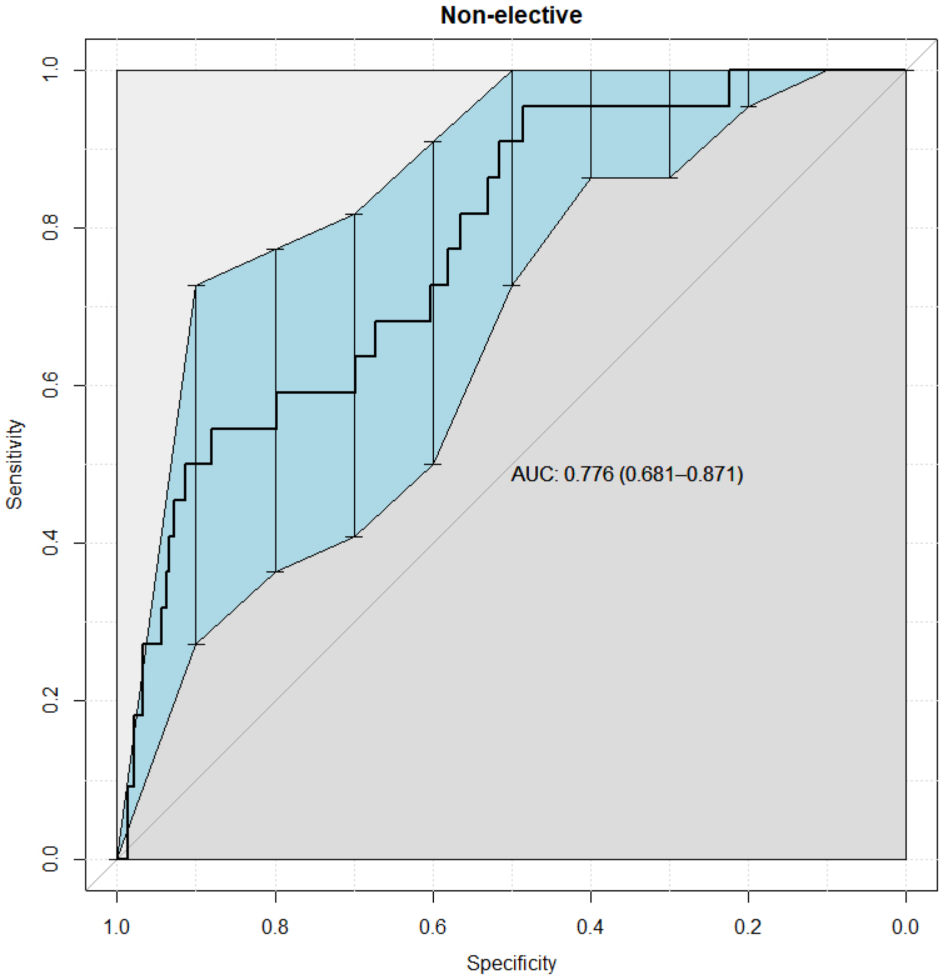

Figure 4a. AUC in the subset of non-elective procedures in the external validation cohort.

67x67mm (300 x 300 DPI)

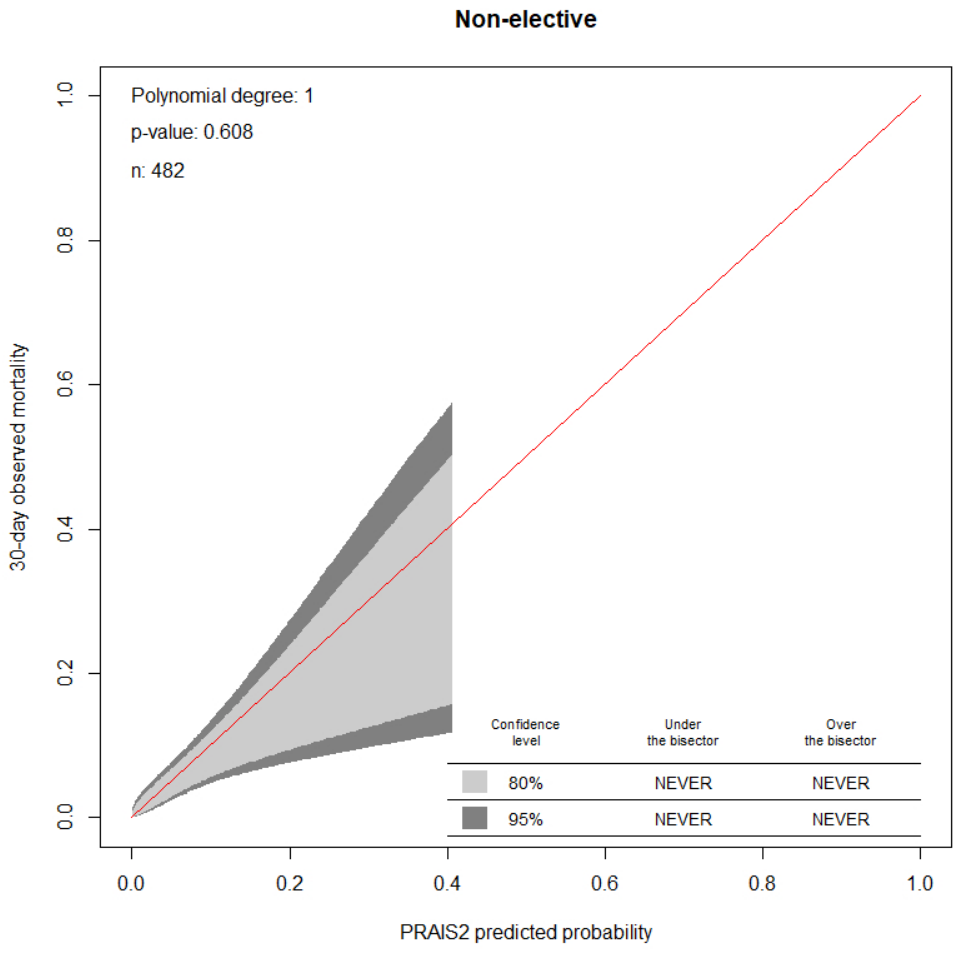

Figure 4b. Calibration belt in the subset of non-elective procedures in the external validation cohort.

67x67mm (300 x 300 DPI)

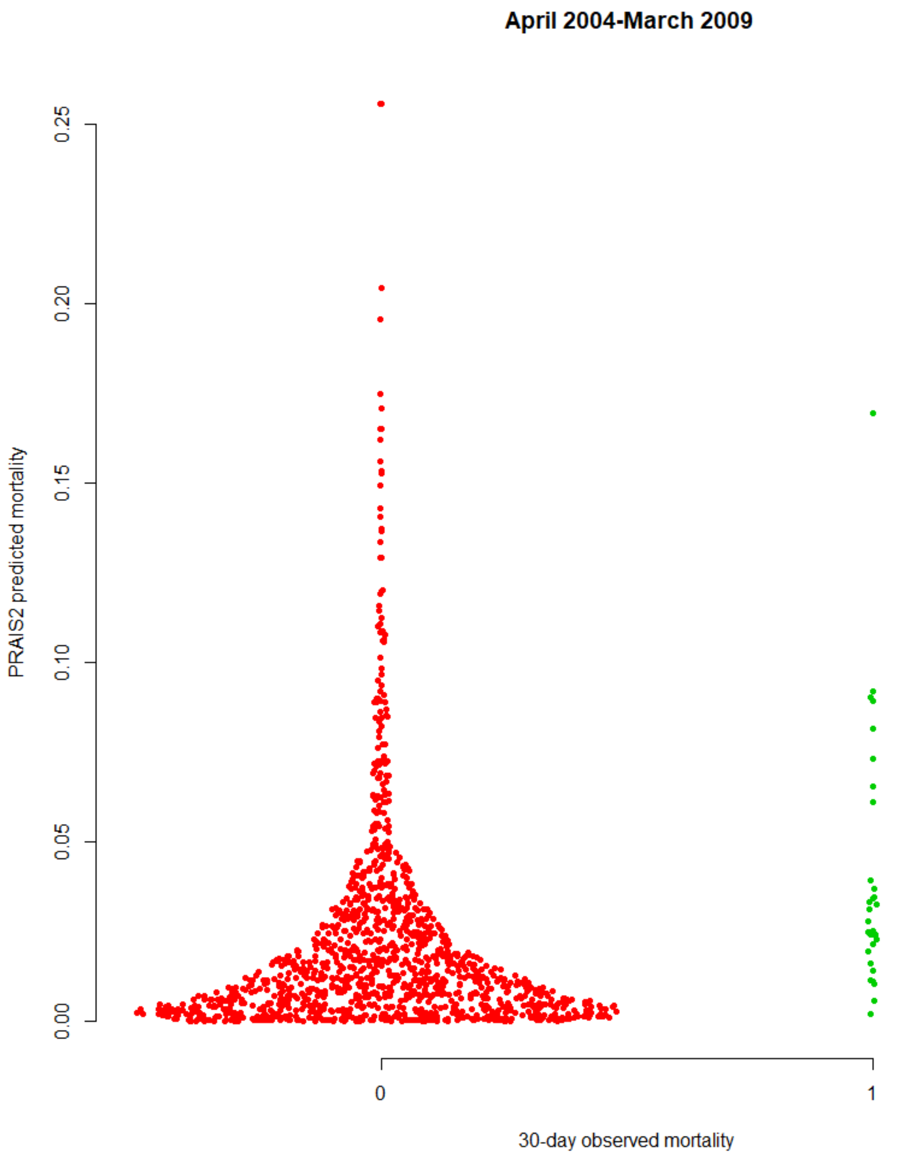
April 2004-March 2009
PRAIS2 predicted mortality
0.00
0.05
0.10
0.15
0.20
0.25
0
1
30-day observed mortality

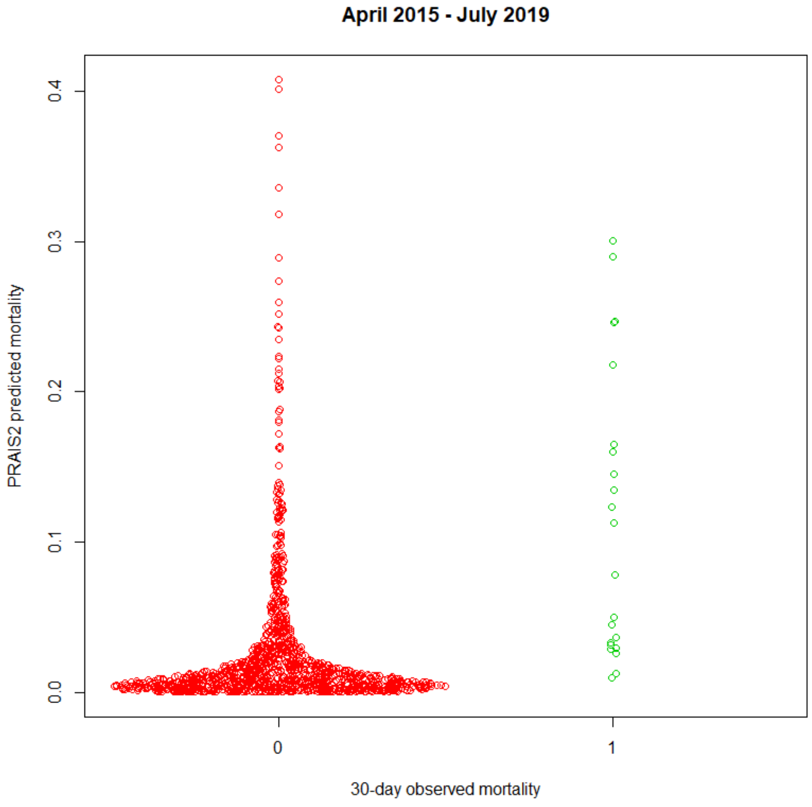
April 2015 - July 2019
PRAIS2 predicted mortality
0.0
0.1
0.2
0.3
0.4
0
1
30-day observed mortality

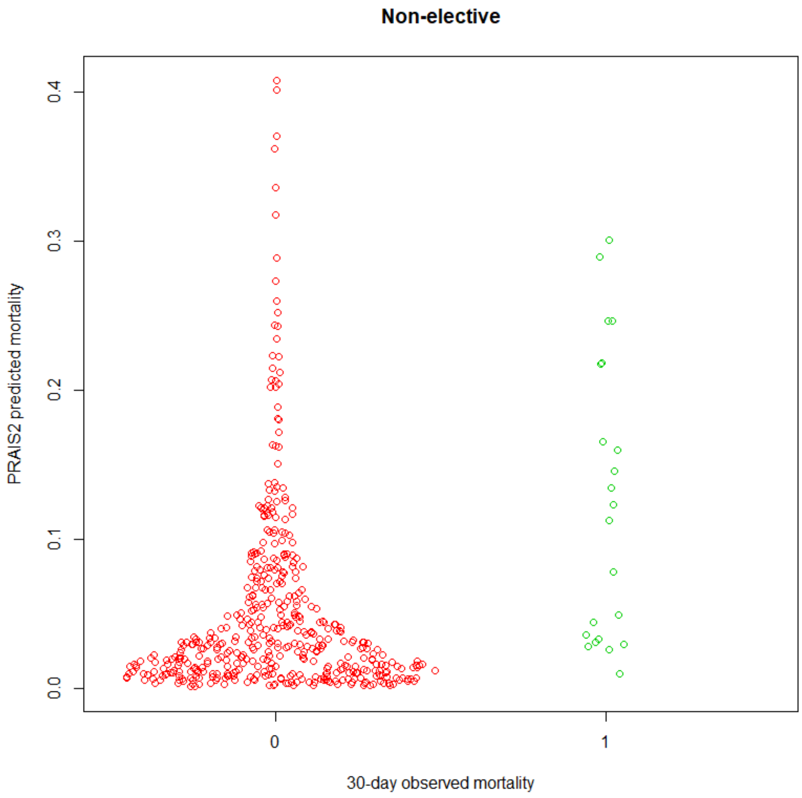
Non-elective
PRAIS2 predicted mortality
0.0
0.1
0.2
0.3
0.4
0
1
30-day observed mortality

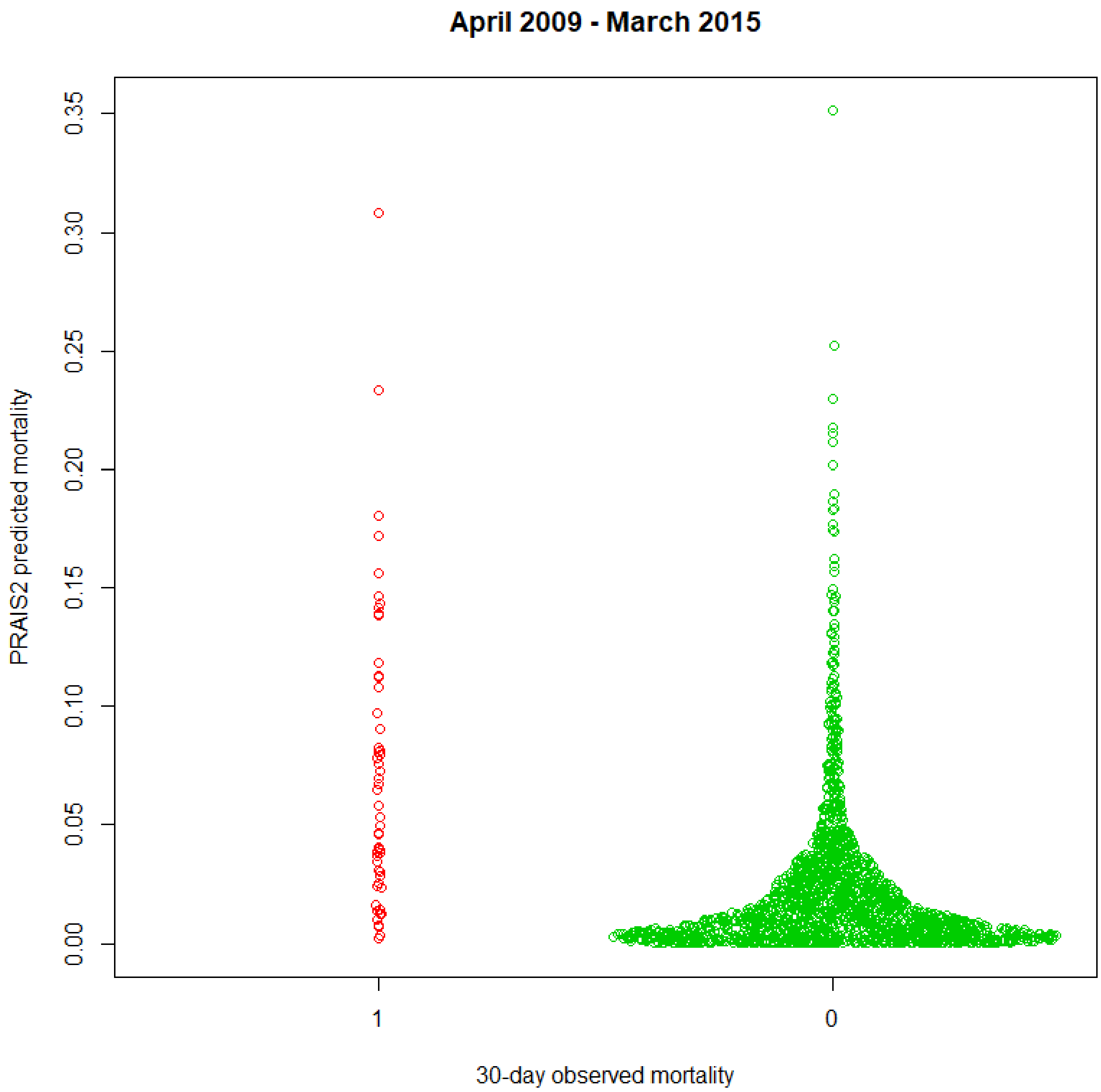
April 2009 - March 2015
PRAIS2 predicted mortality
0.00
0.05
0.10
0.15
0.20
0.25
0.30
0.35
1
0
30-day observed mortality

Supplementary Equation 1. The final recalibrated model

$$Z = -0.229 - 0.439 \times \sqrt{agep} + 0.336 \times age - 1.808 \times \sqrt{weightp} + 0.088 \times weight + \sum coef$$

For peer review only

Supplementary table 1

Diagnoses Group	
1	HLHS; Truncus arteriosus; Pulmonary atresia and IVS
2	Functionally UVH; Pulmonary atresia and VSD
3	TGA þ VSD/DORV-TGA; Interrupted aortic arch
4	PDA
5	Miscellaneous primary congenital diagnosis; Tricuspid valve abnormality (including Ebstein's); TAPVC; No diagnosis given
6	Acquire
7	AVSD; Fallot/DORV Fallot
8	Aortic valve stenosis (isolated); Mitral valve abnormality; Miscellaneous congenital terms
9	TGA + IVS
10	Aortic arch obstruction þ VSD/ASD; Pulmonary stenosis
11	Subaortic stenosis (isolated); Aortic regurgitation; VSD; ASD; Arrhythmia
Procedure diagnose group	
1	Norwood Procedure (Stage 1); HLHS hybrid approach

For peer review only

2	TAPVC repair + arterial shunt; Truncus and interruption repair; Truncus arteriosus repair; Interrupted aortic arch repair; Arterial switch + aortic arch obstruction repair (with-without VSD closure)
3	Arterial shunt
4	Repair of total anomalous pulmonary venous connection; Arterial switch + VSD closure; Isolated pulmonary artery band
5	PDA ligation (surgical)
6	Arterial switch (for isolated transposition); Isolated coarctation/hypoplastic aortic arch repair; Aortopulmonary window repair
7	Senning or Mustard procedure; Ross-Konno procedure; Mitral valve replacement; Pulmonary vein stenosis procedure; Pulmonary atresia VSD repair; Tetralogy with absent pulmonary valve repair; Unifocalization procedure (with/without shunt)
8	Heart transplant; Tricuspid valve replacement; Aortic valve repair; Pulmonary valve replacement; Aortic root replacement (not Ross);Cardiac conduit replacement; Isolated RV to PA conduit construction; Tricuspid valve repair
9	Multiple VSD closure; Atrioventricular septal defect and tetralogy repair; Cor triatriatum repair; Supravalvar aortic stenosis repair; Rastelli-REV procedure
10	Bidirectional cavopulmonary shunt
11	Atrioventricular septal defect (complete) repair
12	Fontan procedure
13	Aortic valve replacement: Ross; Subvalvar aortic stenosis repair; Mitral valve repair; Sinus venosus ASD and/or PAPVC repair
14	Atrioventricular septal defect (partial) repair; Tetralogy of Fallot–type DORV repair; Vascular ring procedure
15	Anomalous coronary artery repair; Aortic valve replacement: non-Ross; ASD repair; VSD repair
20	Cardiac no specific procedure

For peer review only

Acquired Comorbidity	Pulmonary embolism; Secondary systemic hypertension; Systemic hypertension; Primary (essential) systemic hypertension; Systemic hypertension due to aortic arch obstruction; Persistent pulmonary hypertension of the new-born; Necrotising enterocolitis; Meconium aspiration; Pre-procedural coagulation disorder; Pre-procedural renal failure or Pre-procedural renal failure requiring dialysis; Pre-procedural septicaemia; Pre-procedural neurological impairment; Preprocedural cerebral abnormality on imaging; Pre-procedural tracheostomy; Preprocedural seizures; Preprocedural respiratory syncytial virus (RSV) infection; Preprocedural necrotising enterocolitis: treated medically or surgically; Psychomotor developmental delay; Brain Abscess; Cerebrovascular accident (stroke); Anoxic-ischaemic encephalopathy; Hyperthyroidism; Diabetes mellitus or Diabetes mellitus requiring insulin; Meningitis; Kidney failure; Empyema; Lower respiratory tract infection; Lung disease; Asthma; Acquired bronchial disease; Airway disease; Diaphragm disorder: acquired; Diaphragm paralysis; Oesophageal disorder
Additional cardiac risk factor	Ventricular dyssynchrony; Arrhythmogenic right ventricular cardiomyopathy; Right or Left ventricular dysfunction; Ventricular myocardial noncompaction cardiomyopathy; Infectious myocarditis; Drug induced heart muscle disease; Trypanosomal myocarditis (Chagas' disease); Myocardial failure in end stage congenital heart disease; Heart muscle disease in cardiac rejection; Nutritional heart muscle disease; Heart muscle disease in infant of diabetic mother; Heart muscle disease in collagen vascular/ connective tissue disorder; Myocarditis; Ischaemic heart disease; Cardiomyopathy; Restrictive cardiomyopathy; Idiopathic restrictive cardiomyopathy; Endocardial fibroelastosis; Infiltrative cardiomyopathy; Hypertrophic cardiomyopathy; Dilated cardiomyopathy; Pulmonary arterial hypertension; Idiopathic (primary) pulmonary hypertension; Pulmonary vascular disease; Irreversible pulmonary vascular disease due to congenital heart disease (Eisenmenger Syndrome); Secondary pulmonary hypertension; Pulmonary hypertension due to congenital systemic-to-pulmonary shunt; Elevated lung resistance for biventricular repair (> 6 Wood units); Elevated lung resistance for heart transplant (> 4 Wood units); Elevated lung resistance for univentricular repair (> 2 Wood units); Transient myocardial ischaemia; Myocardial infarction; Acute myocardial infarction; Pre-procedural pulmonary hypertension; Preprocedural myocardial infarction; Preprocedural pulmonary hypertension (pulmonary pressure more than or equal to systemic pressure): echo data or catheter data; Residual pulmonary hypertension after relief of L to R shunt

For peer review only

Congenital comorbidity	Visceral heterotaxy (abnormal arrangement thoraco-abdominal organs); Position or morphology of thoraco-abdominal organs abnormal; Lung anomaly; Functionally congenital single lung; Tracheobronchial anomaly; Intestines malrotated; Hereditary disorder associated with heart disease; Trisomy 18 - Edwards syndrome; Trisomy 13 - Pataus syndrome; 45XO - Turners syndrome; 22q11 microdeletion - CATCH 22; Syndrome/association with cardiac involvement; DiGeorge sequence; Friedreich's ataxia; Marfan syndrome; Noonan syndrome; Pompe's disease: glycogen storage disease type IIa; Tuberous sclerosis; Williams syndrome (infantile hypercalcaemia); Fetal rubella syndrome; Duchenne's muscular dystrophy; Muscular dystrophy; Ehlers-Danlos syndrome; Alagille syndrome: arteriohepatic dysplasia; Non-cardiac abnormality associated with heart disease; Non-cardiothoracic / vascular abnormality (DESCRIBE); Cystic fibrosis; Diaphragmatic hernia; Tracheo-oesophageal fistula; Omphalocoele; Duodenal stenosis/atresia; Sickle cell disease; Renal abnormality; Congenital coagulation disorder; Thoracic / mediastinal abnormality; Microcephaly; Choanal atresia; Tracheobronchial malacia; Hypothyroidism; Cerebral anomaly; Connective tissue disease; Kyphoscoliosis; Cleft lip / palate; Loeys-Dietz Syndrome (transforming growth factor beta receptor (TGFBR) gene); Von Willebrand disease; Maternally derived fetal disease or syndrome associated with heart disease; Major anomaly of gastrointestinal system; Multiple congenital malformations; Tracheal stenosis or disease
Severity of illness	Pre-procedural shock; Pre-procedural acidosis; Pre-procedural mechanical ventilatory or circulatory support; Preprocedural shock at time of surgery (persistent); Preprocedural cardiopulmonary resuscitation (< 48 hours); Cardiac Arrest; Respiratory failure;

ASD = atrial septal defect; AVSD = atrioventricular septal defect; DORV = double outlet right ventricle; HLHS = hypoplastic left heart syndrome; IVS = intact ventricular septum; PA = pulmonary artery; PAPVC = partial anomalous pulmonary venous connection; PDA = patent ductus arteriosus; TAPVC = total anomalous pulmonary venous connection; REV = reparation a letage ventriculaire; RV = right ventricle; TGA = transposition of the great arteries; UVH = univentricular heart; VSD = ventricular septal defect.

For peer review only

For peer review only

Supplementary Table 2

2a. Hosmer and Lemeshow goodness of fit groups, external validation cohort 1

PRAIS2 categories	Predicted survival	Predicted mortality	Observed survival	Observed mortality
[3.61e-05,0.000927]	132.9388	0.06122354	133	0
[0.000927,0.00249]	132.7736	0.22635598	132	1
[0.00249,0.00482]	132.5155	0.48445930	133	0
[0.00482,0.00802]	132.1368	0.86323928	131	2
[0.00802,0.0115]	131.7083	1.29173262	130	3
[0.0115,0.0164]	131.1824	1.81763537	131	2
[0.0164,0.0222]	130.4860	2.51397487	131	2
[0.0222,0.0308]	129.5187	3.48125996	126	7
[0.0308,0.0463]	128.0397	4.96026202	125	8
[0.0463,0.256]	121.2271	11.77293767	127	6

For peer review only

2b. Hosmer and Lemeshow goodness of fit groups, external validation cohort 2

PRAIS2 categories	Predicted survival	Predicted mortality	Observed survival	Observed mortality
[0.000131,0.00125]	118.9273	0.07268051	119	0
[0.00125,0.00273]	118.7637	0.23632366	119	0
[0.00273,0.00493]	117.5494	0.45061926	118	0
[0.00493,0.00709]	118.2901	0.70991743	119	0
[0.00709,0.0101]	117.9783	1.02166557	118	1
[0.0101,0.0145]	116.6018	1.39819713	117	1
[0.0145,0.0206]	116.9503	2.04966950	119	0
[0.0206,0.0339]	114.7955	3.20445956	113	5
[0.0339,0.0743]	113.0781	5.92186013	116	3
[0.0743,0.408]	101.4640	17.53604435	106	13

For peer review only

2c. Hosmer and Lemeshow goodness of fit groups, overlapping cohorts

PRAIS2 categories	Predicted survival	Predicted mortality	Observed survival	Observed mortality
[4.29e-05,0.000909]	179.9167	0.08334853	180	0
(0.000909,0.00231]	178.7233	0.27665267	179	0
(0.00231,0.00444]	178.4120	0.58804885	176	3
(0.00444,0.00751]	178.9427	1.05730682	178	2
(0.00751,0.0108]	177.3823	1.61770441	178	1
(0.0108,0.016]	176.6366	2.36336224	175	4
(0.016,0.0231]	176.4888	3.51120063	179	1
(0.0231,0.0332]	174.0463	4.95370602	173	6
(0.0332,0.0548]	171.6407	7.35925742	168	11
(0.0548,0.351]	161.5016	18.49839298	152	28

For peer review only

2d. Hosmer and Lemeshow goodness of fit groups, in non-elective subset

PRAIS2 categories	Predicted survival	Predicted mortality	Observed survival	Observed mortality
[0.00109,0.00594]	48.81584	0.1841625	49	0
[0.00594,0.00946]	47.64347	0.3565252	48	0
[0.00946,0.0138]	47.45516	0.5448356	47	1
[0.0138,0.0201]	47.19803	0.8019745	48	0
[0.0201,0.0286]	46.82881	1.1711897	46	2
[0.0286,0.0382]	46.45216	1.5478412	44	4
[0.0382,0.0542]	45.84400	2.1560041	46	2
[0.0542,0.0818]	44.74003	3.2599674	47	1
[0.0818,0.126]	43.06033	4.9396731	46	2
[0.126,0.408]	38.43469	10.5653107	39	10

For peer review only

TRIPOD Checklist: Prediction Model Validation

Section/Topic	Item	Checklist Item	Page
**Title and abstract**			
Title	1	Identify the study as developing and/or validating a multivariable prediction model, the target population, and the outcome to be predicted.	yes
Abstract	2	Provide a summary of objectives, study design, setting, participants, sample size, predictors, outcome, statistical analysis, results, and conclusions.	yes
**Introduction**			
Background and objectives	3a	Explain the medical context (including whether diagnostic or prognostic) and rationale for developing or validating the multivariable prediction model, including references to existing models.	yes
	3b	Specify the objectives, including whether the study describes the development or validation of the model or both.	yes
**Methods**			
Source of data	4a	Describe the study design or source of data (e.g., randomized trial, cohort, or registry data), separately for the development and validation data sets, if applicable.	yes
	4b	Specify the key study dates, including start of accrual; end of accrual; and, if applicable, end of follow-up.	yes
Participants	5a	Specify key elements of the study setting (e.g., primary care, secondary care, general population) including number and location of centres.	yes
	5b	Describe eligibility criteria for participants.	yes
	5c	Give details of treatments received, if relevant.	yes
Outcome	6a	Clearly define the outcome that is predicted by the prediction model, including how and when assessed.	yes
	6b	Report any actions to blind assessment of the outcome to be predicted.	no
Predictors	7a	Clearly define all predictors used in developing or validating the multivariable prediction model, including how and when they were measured.	yes
	7b	Report any actions to blind assessment of predictors for the outcome and other predictors.	no
Sample size	8	Explain how the study size was arrived at.	yes
Missing data	9	Describe how missing data were handled (e.g., complete-case analysis, single imputation, multiple imputation) with details of any imputation method.	yes
Statistical analysis methods	10c	For validation, describe how the predictions were calculated.	yes
	10d	Specify all measures used to assess model performance and, if relevant, to compare multiple models.	yes
	10e	Describe any model updating (e.g., recalibration) arising from the validation, if done.	NA
Risk groups	11	Provide details on how risk groups were created, if done.	NA
Development vs. validation	12	For validation, identify any differences from the development data in setting, eligibility criteria, outcome, and predictors.	yes
**Results**			
Participants	13a	Describe the flow of participants through the study, including the number of participants with and without the outcome and, if applicable, a summary of the follow-up time. A diagram may be helpful.	yes
	13b	Describe the characteristics of the participants (basic demographics, clinical features, available predictors), including the number of participants with missing data for predictors and outcome.	yes
	13c	For validation, show a comparison with the development data of the distribution of important variables (demographics, predictors and outcome).	yes
Model performance	16	Report performance measures (with CIs) for the prediction model.	yes
Model-updating	17	If done, report the results from any model updating (i.e., model specification, model performance).	NA
**Discussion**			
Limitations	18	Discuss any limitations of the study (such as nonrepresentative sample, few events per predictor, missing data).	yes
Interpretation	19a	For validation, discuss the results with reference to performance in the development data, and any other validation data.	yes
	19b	Give an overall interpretation of the results, considering objectives, limitations, results from similar studies, and other relevant evidence.	yes
Implications	20	Discuss the potential clinical use of the model and implications for future research.	yes
**Other information**			
Supplementary information	21	Provide information about the availability of supplementary resources, such as study protocol, Web calculator, and data sets.	yes
Funding	22	Give the source of funding and the role of the funders for the present study.	yes

We recommend using the TRIPOD Checklist in conjunction with the TRIPOD Explanation and Elaboration document.

For peer review only

TRIPOD Checklist: Prediction Model Validation

	Page
Title	1
Abstract	2
Key question	3
Introduction	3-5
Methods	5-7
Results	7-12
Discussion	13-15
Acknowledge	15
Funding	15-16
Conflict	16
Figure legend	16
References	17-18
Contributorship statement	18

For peer review only

BMJ Open

EXTERNAL VALIDATION OF THE IMPROVING PARTIAL RISK ADJUSTMENT IN SURGERY (PRAIS2) MODEL FOR 30-DAY MORTALITY AFTER PAEDIATRIC CARDIAC SURGERY

Journal:	*BMJ Open*
Manuscript ID	bmjopen-2020-039236.R1
Article Type:	Original research
Date Submitted by the Author:	28-Sep-2020
Complete List of Authors:	Cocomello, Lucia; University of Bristol, MRC Integrative Epidemiology Unit<br>Caputo, Massimo; University of Bristol, Bristol Heart Institute<br>Cornish, Rosie; University of Bristol, School of Social and Community Medicine<br>Lawlor, Deborah; Department of Social Medicine, University of Bristol, MRC Integrative Epidemiology Unit
**Primary Subject Heading**:	Cardiovascular medicine
Secondary Subject Heading:	Cardiovascular medicine, Paediatrics, Surgery
Keywords:	Congenital heart disease < CARDIOLOGY, Paediatric cardiology < CARDIOLOGY, Paediatric cardiac surgery < PAEDIATRIC SURGERY, Paediatric cardiothoracic surgery < PAEDIATRIC SURGERY, Cardiothoracic surgery < SURGERY

SCHOLARONE™
Manuscripts

I, the Submitting Author has the right to grant and does grant on behalf of all authors of the Work (as defined in the below author licence), an exclusive licence and/or a non-exclusive licence for contributions from authors who are: i) UK Crown employees; ii) where BMJ has agreed a CC-BY licence shall apply, and/or iii) in accordance with the terms applicable for US Federal Government officers or employees acting as part of their official duties; on a worldwide, perpetual, irrevocable, royalty-free basis to BMJ Publishing Group Ltd ("BMJ") its licensees and where the relevant Journal is co-owned by BMJ to the co-owners of the Journal, to publish the Work in this journal and any other BMJ products and to exploit all rights, as set out in our licence.

The Submitting Author accepts and understands that any supply made under these terms is made by BMJ to the Submitting Author unless you are acting as an employee on behalf of your employer or a postgraduate student of an affiliated institution which is paying any applicable article publishing charge ("APC") for Open Access articles. Where the Submitting Author wishes to make the Work available on an Open Access basis (and intends to pay the relevant APC), the terms of reuse of such Open Access shall be governed by a Creative Commons licence – details of these licences and which Creative Commons licence will apply to this Work are set out in our licence referred to above.

Other than as permitted in any relevant BMJ Author's Self Archiving Policies, I confirm this Work has not been accepted for publication elsewhere, is not being considered for publication elsewhere and does not duplicate material already published. I confirm all authors consent to publication of this Work and authorise the granting of this licence.

For peer review only

EXTERNAL VALIDATION OF THE IMPROVING PARTIAL RISK ADJUSTMENT IN SURGERY (PRAIS-2) MODEL FOR 30-DAY MORTALITY AFTER PAEDIATRIC CARDIAC SURGERY

Corresponding Author:

Lucia Cocomello,
MRC Integrative Epidemiology Unit,
Oakfield House, Oakfield Grove, Bristol, BS8 2BN, United Kingdom
nn18747@bristol.ac.uk;

Massimo Caputo
Bristol Heart Institute,
Terrell St, Bristol, BS2 8 ED, United Kingdom
M.Caputo@bristol.ac.uk;

Rosie Cornish
MRC Integrative Epidemiology Unit,
Oakfield House, Oakfield Grove, Bristol, BS8 2BN, United Kingdom;
Population Health Sciences, Bristol Medical School, University of Bristol, United Kingdom.
rosie.cornish@bristol.ac.uk

Deborah A. Lawlor
MRC Integrative Epidemiology Unit at the University of Bristol,
Oakfield House, Oakfield Grove, Bristol, BS8 2BN, United Kingdom;
Population Health Sciences, Bristol Medical School, University of Bristol, United Kingdom;
Bristol NIHR Biomedical Research Centre.
d.a.lawlor@bristol.ac.uk

KEYWORDS: congenital heart disease, paediatric cardiac surgery, risk prediction

Word count:3165

For peer review only

ABSTRACT

Objective: Risk stratification in paediatric patients undergoing heart surgery remains a challenge. The improving partial risk adjustment in surgery (PRAIS-2) is a risk model predicting 30-day mortality which has been recently developed and validated using a UK-based cohort from April 2009-March 2015. We aimed to perform an independent temporal external validation to explore its generalisability and clinical utility.

Methods: PRAIS-2 validation was carried out using a two temporally different single centre (Bristol, UK) cohorts: Cohort 1 surgery undertaken from April 2004 to March 2009 and Cohort 2 from April 2015 to July 2019. For each subject PRAIS-2 score was calculated according to the original formula. PRAIS-2 performance was assessed in terms of discrimination by means of ROC curve analysis and calibration by using the calibration belt method.

Results: A total of 1352 (2004-2009) and 1197 (2015-2019) paediatric cardiac surgical procedures were included in the Cohort 1 and Cohort 2, respectively (median age at the procedure 6.3 and 7.1 months). PRAIS-2 score showed excellent discrimination for both cohorts (AUC 0.72 (95%CI: 0.65 to 0.80) and 0.88 (95%CI: 0.82 to 0.93), respectively). While PRAIS-2 was only marginally calibrated in Cohort 1, with a tendency to underestimate risk in low risk and overestimate risk in high risk procedures (P-value = 0.033), validation in Cohort 2 showed good calibration with the 95% confidence belt containing the bisector for predicted mortality (P-value = 0.143). We also observed good prediction accuracy in the non-elective procedures (N = 483; AUC 0.78 (95%CI 0.68 to 0.87); Calibration belt containing the bisector (P-value=0.589).

Conclusions: In a single centre UK-based cohort, PRAIS-2 showed excellent discrimination and calibration in predicting 30-day mortality in paediatric cardiac surgery including in those undergoing non-elective procedures. Our results support a wider adoption of PRAIS-2 score in the clinical practice.

For peer review only - http://bmjopen.bmj.com/site/about/guidelines.xhtml

Strengths and limitations of this study

- A strength of the present study is that data were prospectively collected as part of the UK National Congenital Heart Disease Audit and as such they have undergone continuous and inclusive systematic validation that includes the review of a sample of case notes by external auditors to ensure coding accuracy.
- We used a recently proposed method (calibration belt) which does not require patients to be categorised according to risk percentile but rather provides a risk function across all risk value with 95% CI providing a measure of uncertainty / imprecision
- A key limitation of this study is that the sample size is relatively small and considerably smaller than the cohort used to develop PRAIS-2

For peer review only

INTRODUCTION

Congenital heart diseases (CHD) are the most common birth defect affecting between 6-8 per 1000 of live born children in middle and high income countries.[1 2] Around 5000 paediatric cardiac surgical procedures are performed each year in the United Kingdom. Despite overall mortality being low (3%), there is notable variation in mortality rates between centres. Mortality rates, and variation between centres, are monitored by public and healthcare regulatory bodies as part of assessing the quality of care provided by the centres. In order to monitor a centre's quality of care and performance and make fair comparisons between centres in terms of mortality following paediatric cardiac surgery it is essential to take account of differences in case mix across centres.[3] This requires accurate risk stratification. However, CHD includes a large spectrum of diagnoses with a wide range of surgical procedures performed in the context of a relatively small number of patients. These characteristics make risk stratification extremely challenging.

Several methods that aim to provide an objective risk assessment for taking account of case mix when assessing centre performance have been developed. These include consensus based methods, such as the Risk Adjustment for Congenital Heart Surgery-1 Categories (RACHS-1 Categories) [4] and Aristotle,[5] and more recently empirical research based methods, such as The Society of Thoracic Surgeons - European Association for Cardio-Thoracic Surgery Congenital Heart Surgery Mortality Categories (STAT Mortality Categories)[6], the Society of Thoracic Surgeons Congenital Heart Surgery Database (STS-CHSD) Mortality Risk Model for Congenital Cardiac Surgery, [7-9] and the partial risk adjustment in surgery (PRAIS)[10], which has been developed in the UK and proposed for measuring between centre variation in mortality across the UK. The most recently updated version, PRAIS-2[11], was developed to predict 30-day mortality using data from the UK National Congenital Heart Disease Audit (NCHDA).[12] In comparison to PRAIS, PRAIS-2 included more detailed information about acuity, diagnosis and comorbidities and was shown to have better discrimination and calibration than the original PRAIS[13]. However, adoption of PRAIS-2 into clinical practice is

For peer review only

still limited and this has been partially attributed to the lack of external independent validation other than the one presented by the authors in the original paper.[14] That validation used data from the same UK dataset but was undertaken on a temporally independent sub-cohort (Figure 1). Whilst such validation within a study is important, using the same cohort (and same group of investigators) can result in risk of optimism bias, which would tend to exaggerate risk stratification accuracy in comparison to findings from independent cohorts and investigators.[15],[16] Moreover, prediction models can present significant calibration drift due to temporal and geographic differences in case mix, patients characteristics and surgical technique; the impact of this on the performance of PRAIS-2 is unknown.[17 18] Lastly, as the data used to develop PRAIS-2 did not include information on whether the procedures were elective or conducted as non-elective (emergencies, urgency and salvage) it was not possible to determine whether performance was similar in both of these situations. It is not implausible that stratification accuracy will differ between the two.

The purpose of this study was to perform an external independent validation of the PRAIS-2 score in a cohort from a single tertiary paediatric UK centre (in Bristol, South West England). This single UK centre contributed to the cohort in which PRAIS-2 was originally developed, but with data from different time periods than used here. In this study we determine performance discrimination separately in two Bristol Heart Institute cohorts: (i) procedures performed earlier than those used in the initial development and validation of PRAIS-2 (Cohort 1) and (ii) procedures performed after those used in the initial development and validation of PRAIS-2 (Cohort 2). To explore possible calibration drift, we have compared calibration of the model prediction between three Bristol Heart Institute cohorts; procedures undertaken 2004-2009 (Cohort 1), 2009-2015 (Cohort overlapping the dataset included in PRAIS-2 development) and 2015 to 2019 (Cohort 2). In a subsample, we were also able to undertake the first (exploratory) analysis of how well PRAIS-2 performs in those undergoing non-elective procedures.

For peer review only

METHODS

The study complies with the Declaration of Helsinki. This study was approved by the institutional review board (IRB) at the Bristol Heart Institute (reference number 250868). As this analysis came under clinical audit / quality of care assessment and all data were anonymised following the governance criteria of the NHS, the IRB agreed informed consent was not required.

Patient and public involvement

This research was done without Patient and Public Involvement.

Data Source

Data were obtained from the Bristol dataset which is part of the National Congenital Heart Disease Audit (NCHDA) within the National Institute of Cardiovascular Outcomes Research (NICOR). As such the data used here have undergone continuous and inclusive systematic validation, which includes the review of a sample of case notes by external auditors to ensure coding accuracy.[12] **Table 1** summarises the data sources used in this study and their relationship to the UK National data used in the development of PRAIS-2.

For peer review only

Table 1. Flowchart of procedures included in the original PRAIS-2 validation and the current study

<table>
<tr><th></th><th>PRAIS-2

national sample</th><th colspan="2">Bristol cohort</th></tr>
<tr><td>April 2004-March 2009</td><td>None</td><td colspan="2">n= 1352

Fully independent validation Cohort 1 and Exploration of temporal calibration drift</td></tr>
<tr><td>April 2009-March 2014</td><td>n=21838

Original training</td><td colspan="2">n=1515

Geographical and Temporal calibration drift, Overlapping Cohort</td></tr>
<tr><td>April 2014-March 2015</td><td>n=4207

Original external validation</td><td colspan="2">n=309

Combined with 1515 above for geographical and Temporal calibration drift, Overlapping Cohort</td></tr>
<tr><td rowspan="2">April 2015-July 2019</td><td rowspan="2">None</td><td colspan="2">n=1197

Fully independent validation Cohort 2 and Exploration of temporal calibration drift
(With priority data)</td></tr>
<tr><td>Elective n=714</td><td>Non-elective=483

First exploration of whether PRAIS-2 accurately stratifies non-elective procedures</td></tr>
</table>

For peer review only

For peer review only - http://bmjopen.bmj.com/site/about/guidelines.xhtml

The entire (i.e. including all 3 cohorts used in any analyses) Bristol data consisted of 4,886 paediatric cardiac surgical procedures (defined as surgery on the heart or great vessel in patients aged < 16 years old, excluding catheter procedures and trivial/minor procedures) performed between April 2004 and July 2019. Procedures with missing information on mortality (133), one or more of the variables used in the calculation of PRAIS-2 calculation (371) or both (9) were excluded. Procedures with missing information on one or more of the variables used to calculate PRAIS-2 showed a higher rate of 30-day mortality (Supplementary table 1). For those with missing data on one or more of the variables used to calculate PRAIS-2, the variables that were available suggested that they had higher risk profiles than those with no missing variables, both in the whole study data (Supplementary table 1) and when analysed in the three separate temporal cohorts (Supplementary table 2-4). For example, in those with at least one missing variable (N = 371) the proportion with severe illness was 22.9%, whereas in those with no missing variables (N = 4373) it was 7%. The same results in Cohort 1 were 15.5% and 0%, in the overlapping with PRAIS-2 discovery were 0% and 3.3% and in Cohort 2 were 18.5% and 55.6%. The remaining 4373 (90% of the 4886 eligible) procedures were included in the analysis. PRAIS-2 was developed using national data (including that from the Bristol cohort) from patients undergoing procedures between April 2009 and March 2015. In these analyses we use Bristol data from April 2004 to March 2009 (Cohort 1) and April 2015 to July 2019 (Cohort 2) as independent external validation. Whilst we acknowledge that treatments and mortality rates have changed since 2004, and the first validation set may not reflect contemporary practice, we would expect this to primarily affect model calibration; finding good discrimination for this earlier set would support external validity

For peer review only

and generalisability of PRAIS-2. Seeing good discrimination across all time periods would provide valuable evidence about the generalisability of the PRAIS-2 model. In addition, we explored discrimination and calibration in the Bristol cohort that was also included in the national cohort to develop PRAIS-2, April 2009 to March 2015 (Overlapping Cohort). This allows us to explore any evidence of geographical (centre) difference in stratification performance and using all three cohorts to explore the extent of temporal calibration drift.

PRAIS-2 prediction score and its constituent variables

PRAIS-2 score is generated from a transformed logistic regression model of 30-day mortality following cardiac surgery.[13] The model included the following perioperative variables: age, weight, diagnosis, procedure group, type of procedure, whether or not there was definite univentricular heart function, additional cardiac risk factors, acquired comorbidity, congenital comorbidity, severity of illness and an additional coefficient for procedures performed after 2013 (Supplementary Table 1 shows the units or categories of each of these variables). The formula for the PRAIS-2 score (using the function from the logistic regression model) is

$$\frac{1}{1+e^{-z}}$$

Where z is the logistic model function of the nine variables. In all analyses we used the PRAIS-2 model (equation) that was recalibrated by the original authors after their internal validation (see Supplementary Equation 1).

Outcome

For peer review only

The primary outcome was 30-day all-cause mortality. Information on mortality was obtained from the Office for National Statistics (ONS).

Statistical analysis

Continuous data were presented as medians with interquartile ranges (IQR). Categorical variables were presented as counts with percentages. We assessed the discriminative ability of the PRIAS2 model to distinguish between children who did and did not die within 30-days using the area under the receiver operating characteristic curve (AUC) analysis[19] and its calibration by comparing observed to predicted 30-day mortality rates for groups of procedures with different predicted levels of risk.[14 20 21] Model performance in the main analysis (Cohort 1 and Cohort 2) was also evaluated by reporting sensitivity, specificity, positive- and negative-likelihood ratios (LRP and LRN) and the positive and negative predictive values (PPV and NPV) using cut-off values that included 10%, 20%, 80% and 90% above the cut-off.[22]

Overall model calibration was assessed using the calibration belt method. This method is based on a generalisation of Cox's regression modelling where the relation between the logits of the probability predicted by a model and of the event rates observed in a sample is represented by a polynomial function whose coefficients are fitted and its degree is fixed by a series of likelihood-ratio tests.[23] Risk underestimation is suggested if the calibration belt with its 95% confidence interval (CI) is above the bisector (perfect prediction line) while overestimation is indicated by the belt with its 95% CI being below the bisector. The calibration belt method was accompanied by the Hosmer-Lemeshow X^2 goodness of fit test .[24] Comparison of X^2 were used to investigate the extent of calibration drift across different eras. All analyses were performed using R version 3.5.2 and the following packages: givitiR, ROCit, ggplot2, ResourceSelection.

RESULTS

The final study population consisted of 1352 procedures in Cohort 1 (median age [IQR] 6.3[1.4-30.8]), 1197 procedures in Cohort 2 (median age, [IQR] 7.1[2.2-51.0]), which also included 483 non elective procedures, and 1824 procedures in the Overlapping Cohort (median age, [IQR] 5.9[1.3-38.6]) .

Table 2 shows procedures characteristics of the two external validation (Cohort 1 and Cohort 2) and the subgroup, from Cohort 2, who had non-elective procedures, and the Overlapping Cohort .

While the average PRAIS-2 score did not differ across the temporally different cohorts, we noticed a downward shift in mortality rate in the most recent Cohort 2.

For peer review only

Table 2. Procedure characteristics in the Cohort 1, Cohort 2, in the non-elective subset and Overlapping Cohort.

	Independent validation Cohort 1 (April 2004 - March2009)	Independent validation Cohort 2 (April 2015 - July 2019)	Non-elective Cohort 2 subset (April 2015 - July 2019)	Cohort Overlapping with PRAIS-2 cohort (April 2009- March 2015)
**Total number of procedures**	1352	1197	483	1824
**Age, year (median, IQR)**	6.3[1.4-30.8]	7.1[2.2-51.0]	1.7[0.3-5.0]	5.9[1.3-38.6]
**Weight, kg (median, IQR)**	6.1[3.4-12.5]	6.9[3.9-15.5]	3.7[3.1-5.3]	6.0[3.6-13.4]
**Diagnoses group n (%)** [a]				
GROUP 1	71(5.3)	107(8.9)	47(9.7)	154(8.4)
GROUP 2	149(11.0)	163(13.6)	77(15.9)	194(10.6)
GROUP 3	111(8.2)	103(8.6)	65(13.5)	125(6.9)
GROUP 4	218(16.1)	232(19.4)	118(24.4)	222(12.2)
GROUP 5	104(7.7)	122(10.2)	49(10.1)	123(6.7)
GROUP 6	104(7.7)	137(11.4)	45(9.3)	100(5.5)
GROUP 7	176(13.0)	127(10.6)	34(7.0)	312(17.1)
GROUP 8	155(11.5)	105(8.8)	32(6.6)	192(10.5)
GROUP 9	20(1.5)	<5 (<1)	<5 (<1)	25(1.4)
GROUP 10	56(4.1)	14(1.2)	6(1.2)	95(5.2)
GROUP 11	188(13.9)	85(7.1)	8(1.7)	282(15.5)
**Procedure Group n (%)** [a]				
GROUP 1	<5 (<1)	31(2.6)	22(4.6)	28(1.5)
GROUP 2	29(2.1)	11(0.9)	10(2.1)	33(1.8)
GROUP 3	107(7.9)	46(3.8)	34(7.0)	84(4.6)
GROUP 4	99(7.3)	103(8.6)	73(15.1)	114(6.2)
GROUP 5	239(17.7)	228(19.0)	126(26.1)	344(18.9)
GROUP 6	148(10.9)	53(4.4)	36(7.5)	180(9.9)
GROUP 7	21(1.6)	45(3.8)	17(3.5)	50(2.7)
GROUP 8	86(6.4)	100(8.4)	12(2.5)	126(6.9)
GROUP 9	20(1.5)	16(1.3)	5(1.0)	26(1.4)

For peer review only

GROUP 10	36(2.7)	35(2.9)	7(1.4)	70(3.8)
GROUP 11	36(2.7)	19(1.6)	8(1.7)	57(3.1)
GROUP 12	32(2.4)	64(5.3)	1(0.2)	50(2.7)
GROUP 13	87(6.4)	68(5.7)	5(1.0)	103(5.6)
GROUP 14	38(2.8)	43(3.6)	7(1.4)	55(3.0)
GROUP 15	217(16.1)	144(12.0)	11(2.3)	275(15.1)
GROUP 20	156(11.5)	191(16.0)	109(22.6)	229(12.6)
**Bypass n (%)**	903(66.8)	911(76.1)	273(56.5)	1361(74.6)
**UVH category, n (%)**	143(10.6)	219(18.3)	95(19.7)	258(14.1)
**Severity of illness[b], n (%)**	0(0.0)	222(18.5)	203(42.0)	86(4.7)
**Acquired comorbidity[b], n (%)**	9(0.7)	207(17.3)	132(27.3)	94(5.2)
**Additional cardiac risk factors[b], n (%)**	58(4.3)	82(6.9)	56(11.6)	52(2.9)
**Congenital comorbidity[b], n (%)**	173(12.8)	343(28.7)	143(29.6)	274(15.0)
**PRAIS-2 (median, IQR)**	0.0115[0.0037-0.0258]	0.010[0.004-0.027]	0.029 [0.011-0.068]	0.011[0.003-0.027
**30-day mortality, n (%)**	32(2.4)	23(1.9)	22(4.6)	56(3.1)

[a] as the diagnoses groups and procedure groups have lengthy text details, we have not provided them in the table; they can be found in Supplementary Table 5.

[b] Exact number suppressed for disclosure control purposes

[c]Definitions of variables are given in Supplementary Table 5

For peer review only

The predicted risk in the external validation cohorts (Cohort 1 and Cohort 2), the Overlapping Cohort and the non-elective subset were 1.1%, 1.0%, 1.1.% and 2.9% respectively, and the observed mortality was 2.4%, 1.9%, 3.1% and 4.6%, respectively. (Supplementary Figure 1) In the Cohort 2 external validation analysis, the PRAIS-2 score showed good discrimination ability (AUC 0.87; 95%CI 0.82-0.93, Figure 1) and calibration between predicted and observed mortality across all risk categories with the 95% confidence interval of calibration belt containing the bisector (p value=0.14 (Figure 2; Hosmer and Lemeshow goodness of fit test X-squared = 8, df = 8, p-value = 0.5, Supplementary Table 6a). Sensitivity, Specificity, LRP, LRN, PPV and NPB using different cut-off values of the prediction score are reported in Table 3. Assuming safe effective interventions could reduce those identified at high risk of mortality, in this population we would want to correctly identify the majority of those at risk i.e. minimise false negative results by having a highly specific test, and we would want a positive test to have a high probability of identifying those most at risk (PPV). A 10% threshold maximises specificity (0.90) and maximised the PPV (though this is only 0.10). The 20% threshold also has high specificity (0.80) and a similar PPV (0.06) to the 10% threshold.

Table 3: Metrics of model discrimination according to different PRAIS-2 cut-off values in the Cohort 2

	Metrics	Top 90%	Top 80%	Top 20%	Top 10%
**Cohort 2**	**Cut off**	0.001	0.003	0.03	0.07
	**Sensitivity**	1	1	0.69	0.56
	**Specificity**	0.10	0.20	0.80	0.90
	**PPV**	0.02	0.02	0.06	0.10
	**NPV**	1	1	0.99	0.99
	**LRP**	1.11	1.25	3.64	6.20
	**LRN**	0	0	0.37	0.47

LRP= positive--likelihood ratios; LRN= negative-likelihood ratios; PPV= positive predictive values; NPV=negative predictive values

For peer review only

In Cohort 1, PRAIS-2 showed a lower but still good discrimination ability (AUC 0.72 (95%CI: 0.65 to 0.80, Figure 1), compared with Cohort 1, but was poorly calibrated, with a tendency to underestimate risk in low risk procedures and overestimate in high risk procedures (p-value=0.033, Figure 2; Hosmer and Lemeshow goodness of fit test X-squared = 19, df = 8, p-value = 0.02, Supplementary Table 6b). Supplementary Table 7 shows the discrimination metrics for this cohort, with results similar to those found for Cohort 2 and shown in Table 2.

In additional analyses to check that data from Bristol were not notably different to the main UK wide developmental cohort, we showed that the Overlapping Cohort also had good discrimination, with results similar to the whole developmental cohort (AUC 0.82 (95%CI 0.77-0.88, Figure 1). However, the model was only marginally calibrated with a tendency to risk underestimation (Figure 2). Hosmer and Lemeshow goodness of fit test X-squared = 16, df = 8, p-value = 0.04; (Supplementary Table 6c).

Considering calibration drift across all three temporally different cohorts we observe modest improvement across all three cohorts such that the most recent cohort was the best calibrated. Specifically, the Hosmer-Lemeshow test X^2 decreased from 19 in Cohort 1, through 16 in the Overlapping Cohort to 8 in Cohort 2.

In the subgroup of non-elective procedures (a subgroup of Cohort 2 as these data were not available in the other two cohorts), the model showed good discrimination, with AUC 0.78 (95%CI 0.68-0.87, Figure 3) and good calibration, with the bisector contained within the 95% confidence level of the calibration belt. (p-value 0.61; Figure 4 and Hosmer and Lemeshow goodness of fit test, X-squared = 10, df = 8, p-value = 0.3; Supplementary Table 6d). We were not able to conduct the analysis restricted to elective procedures as all but one patients survived to 30-days following elective procedures.

DISCUSSION

Risk prediction models play an important role in current paediatric cardiac surgical practice because they allow meaningful comparison of outcomes between institutions by adjusting for differing case-mix. Moreover, they can also give useful information for surgical decision making, preoperative patient information and quality assurance measures. Before

For peer review only

implementing a risk model in clinical practice, it is important to confirm its prediction ability with external validation.[14 25]

To our knowledge PRAIS-2 has never undergone external validation other than that originally presented by the authors who developed the model. It is important that prediction models are validated by independent researchers, as well as in independent samples, before being adopted into clinical practice because authors evaluating the performance of their own model may tend to be overly optimistic in interpreting results or selective reporting.[16 26] For this reason we conducted an external validation study of PRAIS-2 in two cohorts from the paediatric cardiac surgical procedures cohort at the Bristol Royal Hospital for Children. Our findings show that in these two cohorts, which are independent (of the original cohort used to develop PRAIS-2), the PRAIS-2 model has good discrimination and good calibration in the most recent (Cohort 2) cohort. That calibration was better in Cohort 2 than in the Overlapping Cohort was surprising as we might expect the cohort contributing to the development of PRAIS-2 to be the best calibrated. We speculate that the temporal improvement in calibration across all three cohorts from the same geographical region is likely to reflect changes over time in case mix, patient risk profile and improvement in perioperative care. Whist we saw an improvement in calibration over time the amount of this was modest. However, a further validation of the PRAIS-2 in the coming years would be useful as marked drift could result in important levels of risk over- or under-estimation.[18]

As PRAIS-2 was developed with data from across the UK and in our external validation we have only used data from Bristol we wanted to make sure that prediction in Bristol was broadly consistent with the UK as a whole. We therefore explored the predictive accuracy of PRAIS-2 in the Bristol cohort that contributed to the original PRAIS-2 development and found good discrimination, but the model was only marginally calibrated with a tendency to underestimate risk. It is not uncommon for calibration to differ between cohorts from different regions or over time. We are not able to determine variation in calibration between all of the different centres

For peer review only

that contributed to the original PRAIS-2 development as data were not presented by centre in the original documentation of PRAIS-2 development.[13]

PRAIS-2 development excluded non-elective procedures and so its accuracy in this group, or whether this would be a valuable predictor, is unknown. We demonstrated that PRAIS-2 does have good discrimination and calibration in this higher risk subgroup. However, we acknowledge that our numbers were small for these analyses and this needs further exploration and validation in larger independent samples. We were not able to compare performance between those undergoing elective procedures and those undergoing non-elective procedures as the mortality rate was very low, resulting in only 1 death among those undergoing elective procedures. This marked difference in mortality between elective and non-elective procedures suggests that elective vs non-elective status might be a valuable covariable in PRAIS-2, but large cohorts with these data would be needed to determine this.

A strength of the present study is that data were prospectively collected as part of the NCHDA national audit. A,s such they have undergone continuous and inclusive systematic validation, which includes the review of a sample of case notes by external auditors to ensure coding accuracy.[12] A key limitation of this study is that the sample size is relatively small and considerably smaller than the cohort used to develop PRAIS-2, which included a total of 21838 procedures (combining development and validation). Further, 10.5% of procedures had missing data on mortality and/or variables used to calculate PRAIS-2. These individuals tended to have higher risk profiles and their mortality rates were higher. Unlike the development cohort that included all UK patients, we only have data from one centre located in the South West of England where the population are more affluent and less ethnically mixed in comparison to the UK as a whole. Hence further replication in a larger and more diverse population is necessary.

The Hosmer and Lemeshow goodness of fit test for calibration is widely used in assessing calibration,[27 28] but it provides a statistical test result comparing predicted to observed outcome rates across arbitrary grouping of deciles of risk, which may have limited clinical

For peer review only

applicability. Therefore, we used a recently proposed method (calibration belt) which does not require patients to be categorised but rather compares across the continuous score of risk for our main method of assessing calibration.[29] We also presented Hosmer and Lemeshow goodness of fit test in the supplementary material for comparison with any previous studies.

In conclusion, our study shows good external validity of PRAIS-2 for predicting short-term mortality in paediatric cardiac surgery (the outcome it was developed to predict). We also show preliminary evidence that PRAIS-2 accurately predicts short-term mortality in non-elective procedures. If similar results were found in more diverse external populations, including those outside of the UK, its wider adoption for risk prediction in paediatric patients undergoing cardiac surgery would be appropriate.

Acknowledgments

We thank Mai Baquedano for support with data management

Funding

This study was supported by the British Heart Foundation Accelerator Award (AA/18/7/34219), which funds LC, and the Bristol National Institute of Health Research Biomedical Research Centre. LC, RC, and DAL work in a unit that receives support from the University of Bristol and the UK Medical Research Council (MC_UU_00011/6). DAL is a National Institute of Health Research Senior Investigator (NF-0616-10102). The views expressed in this publication are those of the author(s) and not necessarily those of the UK National Health Service, the National Institute for Health Research or the UK Department of Health and Social Care, or any other funders mentioned here.

Conflict

DAL has received support from several national and international charity and government grants and from Medtronic Ltd and Roche Diagnostics for research unrelated to that presented here. The other authors declare no conflicts of interest.

Data availability

Data may be obtained from a third party and are not publicly available. Data belong to Univeristy Hospital Bristol NHS Trust and as such as protected by confidentiality, data sharing request will need to adhere to trust policy on confidentiality.

For peer review only

Figure legend

Figure 1. ROC curve for PRAIS-2 in the independent Cohort 1,2 and Overlapping Cohort

Figure 2. Calibration belt of PRAIS-2 in the independent Cohort 1,2 and Overlapping Cohort

Figure 3. AUC in the subset of non-elective procedures in the external validation cohort.

Figure 4. Calibration belt in the subset of non-elective procedures in the external validation cohort.

Supplementary figure 1. Sinaplot of PRAIS2 distribution stratified for incidence of 30-day mortality, 0=survivors and 1= not survivors

For peer review only

REFERENCES

1. Hoffman JI, Kaplan S. The incidence of congenital heart disease. *J Am Coll Cardiol* 2002;39(12):1890-900. doi: 10.1016/s0735-1097(02)01886-7 [published Online First: 2002/06/27]
2. van der Linde D, Konings EE, Slager MA, et al. Birth prevalence of congenital heart disease worldwide: a systematic review and meta-analysis. *J Am Coll Cardiol* 2011;58(21):2241-7. doi: 10.1016/j.jacc.2011.08.025 [published Online First: 2011/11/15]
3. Brown KL, Crowe S, Franklin R, et al. Trends in 30-day mortality rate and case mix for paediatric cardiac surgery in the UK between 2000 and 2010. *Open Heart* 2015;2(1) doi: UNSP e000157

10.1136/openhrt-2014-000157
4. Jenkins KJ, Gauvreau K, Newburger JW, et al. Consensus-based method for risk adjustment for surgery for congenital heart disease. *J Thorac Cardiovasc Surg* 2002;123(1):110-8. doi: 10.1067/mtc.2002.119064 [published Online First: 2002/01/10]
5. Lacour-Gayet F, Clarke D, Jacobs J, et al. The Aristotle score for congenital heart surgery. *Semin Thorac Cardiovasc Surg Pediatr Card Surg Annu* 2004;7:185-91. doi: 10.1053/j.pcsu.2004.02.011 [published Online First: 2004/07/31]
6. O'Brien SM, Clarke DR, Jacobs JP, et al. An empirically based tool for analyzing mortality associated with congenital heart surgery. *J Thorac Cardiovasc Surg* 2009;138(5):1139-53. doi: 10.1016/j.jtcvs.2009.03.071 [published Online First: 2009/10/20]
7. Jacobs JP, O'Brien SM, Hill KD, et al. Refining The Society of Thoracic Surgeons Congenital Heart Surgery Database Mortality Risk Model With Enhanced Risk Adjustment for Chromosomal Abnormalities, Syndromes, and Noncardiac Congenital Anatomic Abnormalities. *Ann Thorac Surg* 2019;108(2):558-66. doi: 10.1016/j.athoracsur.2019.01.069 [published Online First: 2019/03/12]
8. Jacobs JP, O'Brien SM, Pasquali SK, et al. The Society of Thoracic Surgeons Congenital Heart Surgery Database Mortality Risk Model: Part 2-Clinical Application. *Ann Thorac Surg* 2015;100(3):1063-8; discussion 68-70. doi: 10.1016/j.athoracsur.2015.07.011 [published Online First: 2015/08/08]
9. O'Brien SM, Jacobs JP, Pasquali SK, et al. The Society of Thoracic Surgeons Congenital Heart Surgery Database Mortality Risk Model: Part 1-Statistical Methodology. *Ann Thorac Surg* 2015;100(3):1054-62. doi: 10.1016/j.athoracsur.2015.07.014 [published Online First: 2015/08/08]
10. Pagel C, Brown KL, Crowe S, et al. A mortality risk model to adjust for case mix in UK paediatric cardiac surgery. Southampton (UK)2013.
11. Rogers L, Brown KL, Franklin RC, et al. Improving Risk Adjustment for Mortality After Pediatric Cardiac Surgery: The UK PRAIS-2 Model. *Ann Thorac Surg* 2017;104(1):211-19. doi: 10.1016/j.athoracsur.2016.12.014 [published Online First: 2017/03/21]
12. Wang T, Chen L, Yang T, et al. Congenital Heart Disease and Risk of Cardiovascular Disease: A Meta-Analysis of Cohort Studies. *J Am Heart Assoc* 2019;8(10):e012030. doi: 10.1161/jaha.119.012030 [published Online First: 2019/05/10]
13. Pagel C, Rogers L, Brown K, et al. Improving risk adjustment in the PRAiS (Partial Risk Adjustment in Surgery) model for mortality after paediatric cardiac surgery and improving public understanding of its use in monitoring outcomes. Southampton (UK)2017.

For peer review only

14. Altman DG, Vergouwe Y, Royston P, et al. Prognosis and prognostic research: validating a prognostic model. *BMJ* 2009;338:b605. doi: 10.1136/bmj.b605 [published Online First: 2009/05/30]
15. Siontis GC, Tzoulaki I, Siontis KC, et al. Comparisons of established risk prediction models for cardiovascular disease: systematic review. *BMJ* 2012;344:e3318. doi: 10.1136/bmj.e3318 [published Online First: 2012/05/26]
16. Ban JW, Stevens R, Perera R. Predictors for independent external validation of cardiovascular risk clinical prediction rules: Cox proportional hazards regression analyses. *Diagn Progn Res* 2018;2:3. doi: 10.1186/s41512-018-0025-6 [published Online First: 2018/02/06]
17. Hickey GL, Blackstone EH. External model validation of binary clinical risk prediction models in cardiovascular and thoracic surgery. *J Thorac Cardiovasc Surg* 2016;152(2):351-5. doi: 10.1016/j.jtcvs.2016.04.023 [published Online First: 2016/05/25]
18. Hickey GL, Grant SW, Murphy GJ, et al. Dynamic trends in cardiac surgery: why the logistic EuroSCORE is no longer suitable for contemporary cardiac surgery and implications for future risk models. *Eur J Cardio-Thorac* 2013;43(6):1146-52. doi: 10.1093/ejcts/ezs584
19. Hanley JA, Mcneil BJ. The Meaning and Use of the Area under a Receiver Operating Characteristic (Roc) Curve. *Radiology* 1982;143(1):29-36. doi: DOI 10.1148/radiology.143.1.7063747
20. Harrell FE, Lee KL, Mark DB. Multivariable prognostic models: Issues in developing models, evaluating assumptions and adequacy, and measuring and reducing errors. *Stat Med* 1996;15(4):361-87. doi: Doi 10.1002/(Sici)1097-0258(19960229)15:4<361::Aid-Sim168>3.0.Co;2-4
21. Mackillop WJ, Quirt CF. Measuring the accuracy of prognostic judgments in oncology. *J Clin Epidemiol* 1997;50(1):21-29. doi: Doi 10.1016/S0895-4356(96)00316-2
22. Usher-Smith JA, Harshfield A, Saunders CL, et al. External validation of risk prediction models for incident colorectal cancer using UK Biobank. *Br J Cancer* 2018;118(5):750-59. doi: 10.1038/bjc.2017.463 [published Online First: 2018/01/31]
23. Nattino G, Finazzi S, Bertolini G. A new calibration test and a reappraisal of the calibration belt for the assessment of prediction models based on dichotomous outcomes. *Stat Med* 2014;33(14):2390-407. doi: 10.1002/sim.6100
24. Fagerland MW, Hosmer DW. A goodness-of-fit test for the proportional odds regression model. *Stat Med* 2013;32(13):2235-49. doi: 10.1002/sim.5645 [published Online First: 2012/10/06]
25. Bleeker SE, Moll HA, Steyerberg EW, et al. External validation is necessary in prediction research: a clinical example. *J Clin Epidemiol* 2003;56(9):826-32. doi: 10.1016/s0895-4356(03)00207-5 [published Online First: 2003/09/25]
26. Collins GS, de Groot JA, Dutton S, et al. External validation of multivariable prediction models: a systematic review of methodological conduct and reporting. *Bmc Med Res Methodol* 2014;14 doi: Artn 40

10.1186/1471-2288-14-40
27. Cook NR. Statistical evaluation of prognostic versus diagnostic models: Beyond the ROC curve. *Clin Chem* 2008;54(1):17-23. doi: 10.1373/clinchem.2007.096529
28. Nashef SAM, Rogues F, Michel P, et al. European system for cardiac operative risk evaluation (EuroSCORE). *Eur J Cardio-Thorac* 1999;16(1):9-13. doi: Doi 10.1016/S1010-7940(99)00134-7
29. Steyerberg EW, Vickers AJ, Cook NR, et al. Assessing the Performance of Prediction Models A Framework for Traditional and Novel Measures. *Epidemiology* 2010;21(1):128-38. doi: 10.1097/EDE.0b013e3181c30fb2

For peer review only

Contributorship Statement:

Lucia Cocomello: Conception and design, data acquisition, analysis and interpretation of data, writing first draft

Massimo Caputo: Critical revision of publication, approval of final publication

Rosie Cornish: Critical revision of publication, approval of final publication

Deborah Lawlor: Design, writing first draft, interpretation of results, supervision

For peer review only

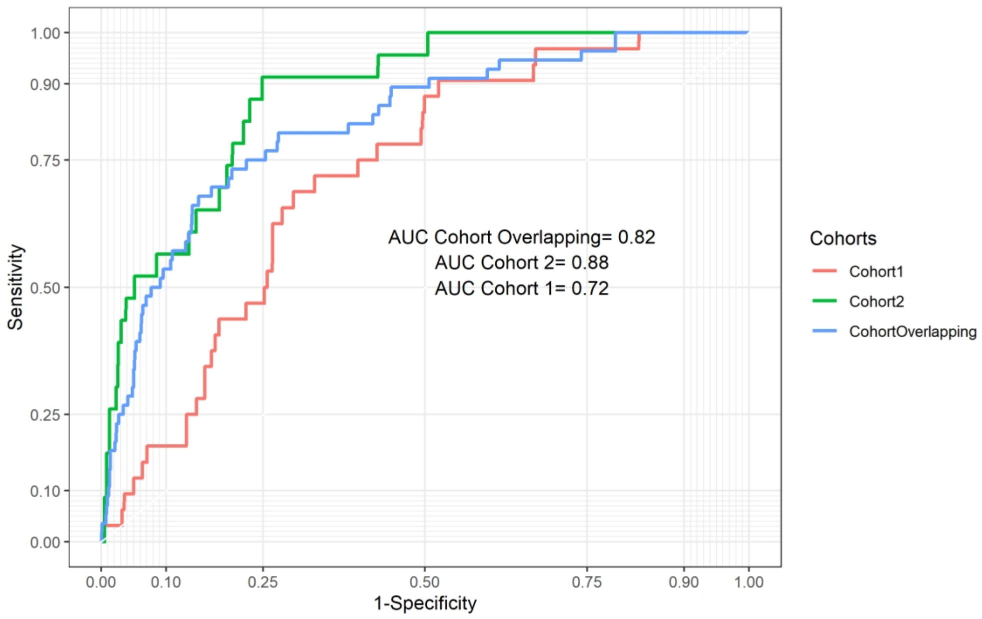

Figure 1. ROC curve for PRAIS-2 in the independent Cohort 1,2 and Overlapping Cohort

203x127mm (300 x 300 DPI)

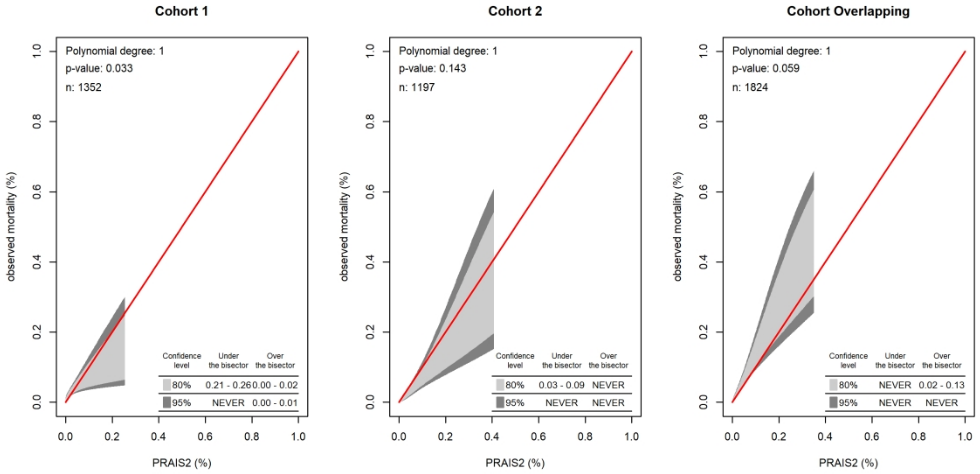

Figure 2. Calibration belt of PRAIS-2 in the independent Cohort 1,2 and Overlapping Cohort

254x127mm (300 x 300 DPI)

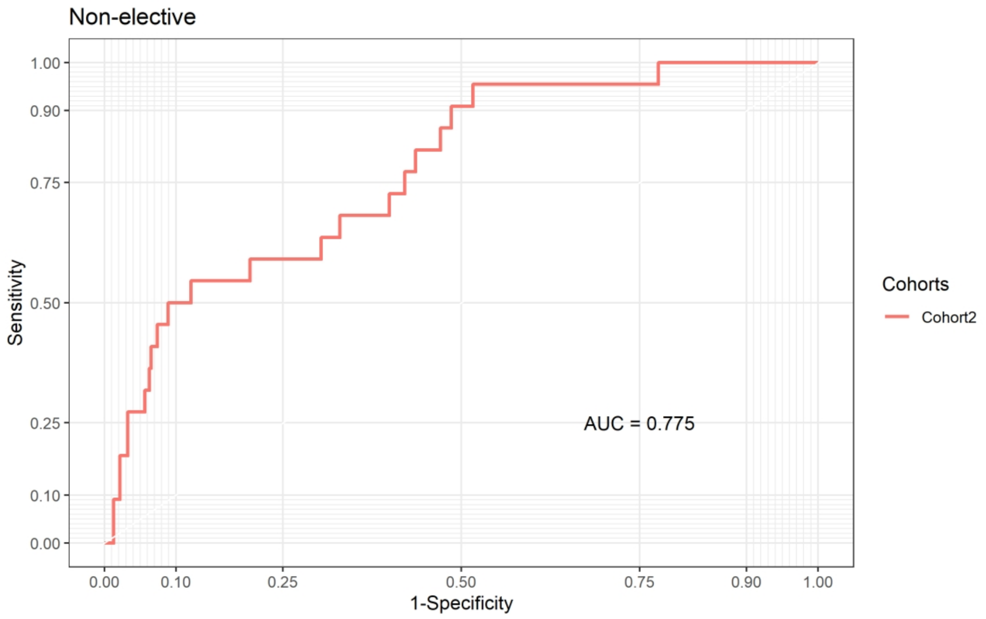

Figure 3. AUC in the subset of non-elective procedures in the external validation cohort.

203x127mm (300 x 300 DPI)

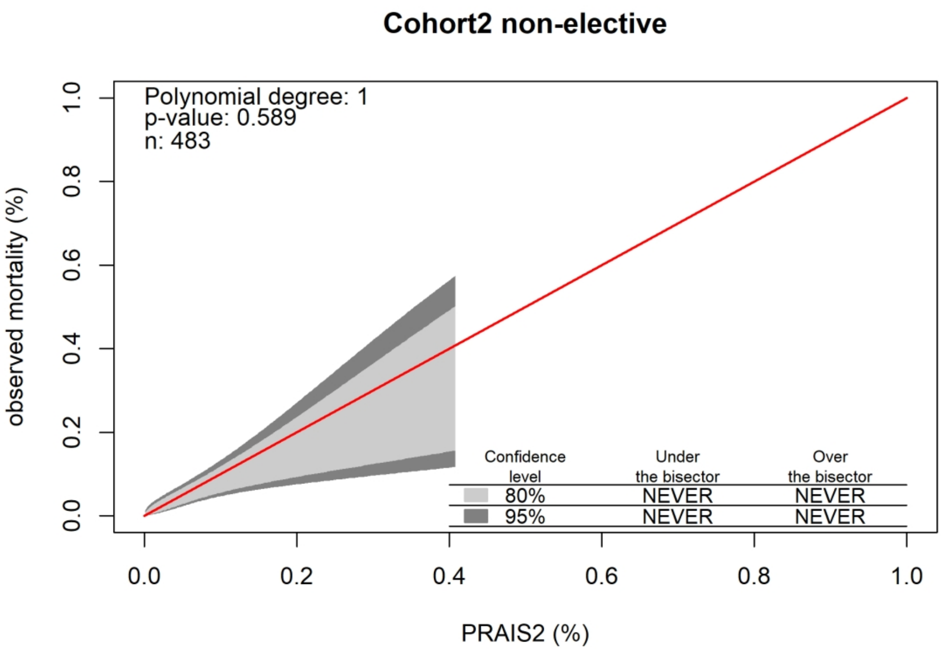

Figure 4. Calibration belt in the subset of non-elective procedures in the external validation cohort.

177x127mm (300 x 300 DPI)

Supplementary Equation 1. The final recalibrated model

$$Z = -0.229 - 0.439\times\sqrt{agep} + 0.336\times age - 1.808\times\sqrt{weightp} + 0.088\times weight + \sum coef$$

For peer review only

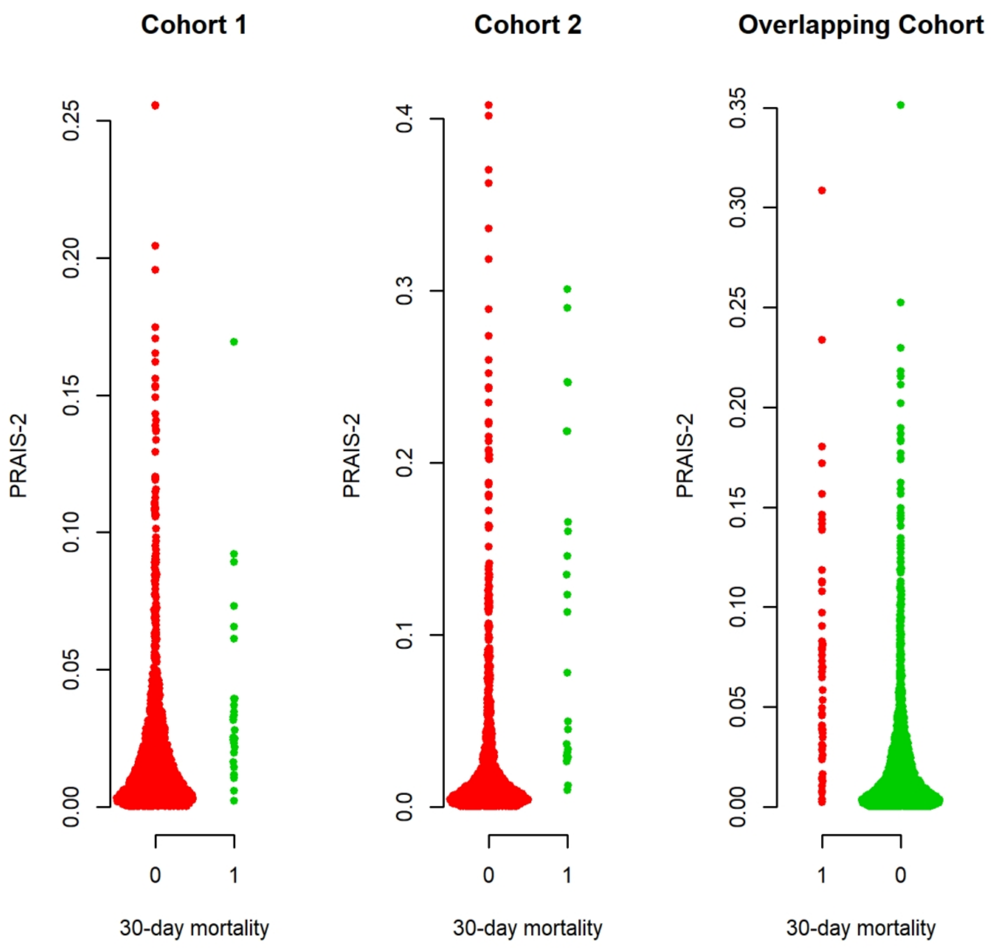

127x127mm (300 x 300 DPI)

Supplementary Table 1 Procedures with missing data on PRAIS2 compared with procedures with complete data

	Non-missing	PRAIS2 Missing
**n**	4373	371
**Age (median, IQR)**	2.55(4.02)	3.39(4.74)
**Diagnoses group n (%)** [a]		
GROUP 1	332(7.6)	51(13.7)
GROUP 2	506(11.6)	46(12.4)
GROUP 3	339(7.8)	27(7.3)
GROUP 4	672(15.4)	33(8.9)
GROUP 5	349(8.0)	36(9.7)
GROUP 6	341(7.8)	52(14.0)
GROUP 7	615(14.1)	21(5.7)
GROUP 8	452(10.3)	18(4.9)
GROUP 9	47(1.1)	1(0.3)
GROUP 10	165(3.8)	6(1.6)
GROUP 11	555(12.7)	21(5.7)
GROUP NA	0(0.0)	59(15.9)
**Procedure Group n (%)** [a]		
GROUP 1	60(1.4)	0(0.0)
GROUP 2	73(1.7)	0(0.0)
GROUP 3	237(5.4)	0(0.0)
GROUP 4	316(7.2)	13(3.5)
GROUP 5	811(18.5)	7(1.9)
GROUP 6	381(8.7)	5(1.3)
GROUP 7	116(2.7)	2(0.5)
GROUP 8	312(7.1)	3(0.8)
GROUP 9	62(1.4)	0(0.0)
GROUP 10	141(3.2)	0(0.0)
GROUP 11	112(2.6)	1(0.3)
GROUP 12	146(3.3)	0(0.0)

For peer review only

GROUP 13	258(5.9)	1(0.3)
GROUP 14	136(3.1)	2(0.5)
GROUP 15	636(14.5)	5(1.3)
GROUP 20	576(13.2)	9(2.4)
GROUP NA	0(0.0)	323(87.1)
**Bypass n (%)**	3175(72.6)	51(13.7)
**Weight (mean, SD)**	11.54(13.86)	14.16(16.78)
**UVH category, n (%)**		
No	3753(85.8)	274(73.9)
Yes	620(14.2)	73(19.7)
NA	0(0.0)	24(6.5)
**Severity of illness[b], n (%)**		
No	4065(93.0)	271(73.0)
Yes	308(7.0)	85(22.9)
NA	0(0.0)	15(4.0)
**Acquired comorbidity[b], n (%)**		
No	4063(92.9)	299(80.6)
Yes	310(7.1)	57(15.4)
NA	0(0.0)	15(4.0)
**Additional cardiac risk factors[b], n (%)**		
No	4181(95.6)	324(87.3)
Yes	192(4.4)	32(8.6)
NA	0(0.0)	15(4.0)
**Congenital comorbidity[b], n (%)**		
No	3583(81.9)	280(75.5)
Yes	790(18.1)	76(20.5)
Na	0(0.0)	15(4.0)
**30-day mortality, n (%)**	111(2.5)	31(8.4)

[a] as the diagnoses groups and procedure groups have lengthy text details, we have not provided them in the table; they can be found in Supplementary Table 5.

[b] Definitions of variables are given in Supplementary Table 5

For peer review only

Supplementary table 2 Procedures with missing data on PRAIS2 compared with procedures with complete data in the Cohort 1

	**Non-missing**	**Missing**
**n**	1352	58
**Age (median, IQR)**	2.39(3.93)	3.59(4.84)
**Diagnoses group n (%)** [a]		
GROUP 1	71(5.3)	2(3.4)
GROUP 2	149(11.0)	5(8.6)
GROUP 3	111(8.2)	5(8.6)
GROUP 4	218(16.1)	5(8.6)
GROUP 5	104(7.7)	6(10.3)
GROUP 6	104(7.7)	9(15.5)
GROUP 7	176(13.0)	1(1.7)
GROUP 8	155(11.5)	3(5.2)
GROUP 9	20(1.5)	0(0.0)
GROUP 10	56(4.1)	1(1.7)
GROUP 11	188(13.9)	2(3.4)
GROUP NA	0(0.0)	19(32.8)
**Procedure Group n (%)** [a]		
GROUP 1	1(0.1)	0(0.0)
GROUP 2	29(2.1)	0(0.0)
GROUP 3	107(7.9)	0(0.0)
GROUP 4	99(7.3)	7(12.1)
GROUP 5	239(17.7)	1(1.7)
GROUP 6	148(10.9)	2(3.4)
GROUP 7	21(1.6)	1(1.7)
GROUP 8	86(6.4)	0(0.0)
GROUP 9	20(1.5)	0(0.0)
GROUP 10	36(2.7)	0(0.0)
GROUP 11	36(2.7)	1(1.7)
GROUP 12	32(2.4)	0(0.0)

For peer review only

GROUP 13	87(6.4)	1(1.7)
GROUP 14	38(2.8)	0(0.0)
GROUP 15	217(16.1)	3(5.2)
GROUP 20	156(11.5)	4(6.9)
GROUP NA	0(0.0)	38(65.5)
**Bypass n (%)**	903(66.8)	19(32.8)
**Weight (mean, SD)**	11.04(13.73)	14.34(16.21)
**UVH category, n (%)**		
No	1209(89.4)	48(82.8)
Yes	143(10.6)	3(5.2)
NA	0(0.0)	7(12.1)
**Severity of illness[b], n (%)**		
NA	0(0.0)	9(15.5)
**Acquired comorbidity[b], n (%)**		
No	1343(99.3)	49(84.5)
Yes	9(0.7)	0(0.0)
NA	0(0.0)	9(15.5)
**Additional cardiac risk factors[b], n (%)**		
No	1294(95.7)	45(77.6)
Yes	58(4.3)	4(6.9)
NA	0(0.0)	9(15.5)
**Congenital comorbidity[b], n (%)**		
No	1179(87.2)	42(72.4)
Yes	173(12.8)	7(12.1)
NA	0(0.0)	9(15.5)
**30-day mortality, n (%)**	32(2.4)	2(3.4)

[a] as the diagnoses groups and procedure groups have lengthy text details, we have not provided them in the table; they can be found in Supplementary Table 5

[b] Definitions of variables are given in Supplementary Table 5

For peer review only

Supplementary table 3 Procedures with missing data on PRAIS2 compared with procedures with complete data in the Cohort 2

	Non-missing	Missing
n	1197	133
**Age (mean, SD)**	2.91(4.22)	3.49(4.76)
**Diagnoses group n (%)** [a]		
GROUP 1	107(8.9)	25(18.8)
GROUP 2	163(13.6)	12(9.0)
GROUP 3	103(8.6)	14(10.5)
GROUP 4	232(19.4)	16(12.0)
GROUP 5	122(10.2)	23(17.3)
GROUP 6	137(11.4)	26(19.5)
GROUP 7	127(10.6)	4(3.0)
GROUP 8	105(8.8)	3(2.3)
GROUP 9	2(0.2)	0(0.0)
GROUP 10	14(1.2)	1(0.8)
GROUP 11	85(7.1)	5(3.8)
GROUP NA	0(0.0)	4(3.0)
**Procedure Group n (%)** [a]		
GROUP 1	31(2.6)	0(0.0)
GROUP 2	11(0.9)	0(0.0)
GROUP 3	46(3.8)	0(0.0)
GROUP 4	103(8.6)	0(0.0)
GROUP 5	228(19.0)	0(0.0)
GROUP 6	53(4.4)	0(0.0)
GROUP 7	45(3.8)	0(0.0)
GROUP 8	100(8.4)	0(0.0)
GROUP 9	16(1.3)	0(0.0)
GROUP 10	35(2.9)	0(0.0)
GROUP 11	19(1.6)	0(0.0)
GROUP 12	64(5.3)	0(0.0)
GROUP 13	68(5.7)	0(0.0)
GROUP 14	43(3.6)	0(0.0)

For peer review only

GROUP 15	144(12.0)	0(0.0)
GROUP 20	191(16.0)	0(0.0)
GROUP NA	0(0.0)	133(100.0)
**Bypass n (%)**	911(76.1)	0(0.0)
**Weight (mean, SD)**	12.57(13.89)	14.31(15.94)
**UVH category, n (%)**		
GROUP 0	978(81.7)	98(73.7)
GROUP 1.2	219(18.3)	31(23.3)
GROUP NA.2	0(0.0)	4(3.0)
**Severity of illness [b], n (%)**		
Yes	222(18.5)	74(55.6)
**Acquired comorbidity [b], n (%)**		
Yes	207(17.3)	43(32.3)
**Additional cardiac risk factors [b], n (%)**		
yes	82(6.9)	17(12.8)
**Congenital comorbidity [b], n (%)**	343(28.7)	41(30.8)
**30-day mortality, n (%)**	23(1.9)	14(10.5)

[a] as the diagnoses groups and procedure groups have lengthy text details, we have not provided them in the table; they can be found in Supplementary Table 5.

[b] Definitions of variables are given in Supplementary Table 5

For peer review only

Supplementary table 4 Procedures with missing data on PRAIS2 compared with procedures with complete data in the Overlapping Cohort

	Non-missing	Missing
**n**	1824	180
**Age (mean, SD)**	2.43(3.93)	3.26(4.71)
**Diagnoses group n (%)** [a]		
GROUP 1	154(8.4)	24(13.3)
GROUP 2	194(10.6)	29(16.1)
GROUP 3	125(6.9)	8(4.4)
GROUP 4	222(12.2)	12(6.7)
GROUP 5	123(6.7)	7(3.9)
GROUP 6	100(5.5)	17(9.4)
GROUP 7	312(17.1)	16(8.9)
GROUP 8	192(10.5)	12(6.7)
GROUP 9	25(1.4)	1(0.6)
GROUP 10	95(5.2)	4(2.2)
GROUP 11	282(15.5)	14(7.8)
GROUP NA	0(0.0)	36(20.0)
**Procedure Group n (%)** [a]		
GROUP 1	28(1.5)	0(0.0)
GROUP 2	33(1.8)	0(0.0)
GROUP 3	84(4.6)	0(0.0)
GROUP 4	114(6.2)	6(3.3)
GROUP 5	344(18.9)	6(3.3)
GROUP 6	180(9.9)	3(1.7)
GROUP 7	50(2.7)	1(0.6)
GROUP 8	126(6.9)	3(1.7)
GROUP 9	26(1.4)	0(0.0)
GROUP 10	70(3.8)	0(0.0)
GROUP 11	57(3.1)	0(0.0)

For peer review only

GROUP 12	50(2.7)	0(0.0)
GROUP 13	103(5.6)	0(0.0)
GROUP 14	55(3.0)	2(1.1)
GROUP 15	275(15.1)	2(1.1)
GROUP 20	229(12.6)	5(2.8)
GROUP NA	0(0.0)	152(84.4)
**Bypass n (%)**	1361(74.6)	32(17.8)
**Weight (mean, SD)**	11.23(13.92)	13.99(17.63)
**UVH category, n (%)**		
No	1566(85.9)	128(71.1)
Yes	258(14.1)	39(21.7)
NA	0(0.0)	13(7.2)
**Severity of illness[b], n (%)**		
No	1738(95.3)	163(90.6)
Yes	86(4.7)	11(6.1)
NA	0(0.0)	6(3.3)
**Acquired comorbidity[b], n (%)**		
No	1730(94.8)	160(88.9)
Yes	94(5.2)	14(7.8)
NA	0(0.0)	6(3.3)
**Additional cardiac risk factors[b], n (%)**		
No	1772(97.1)	163(90.6)
Yes	52(2.9)	11(6.1)
NA	0(0.0)	6(3.3)
**Congenital comorbidity[b], n (%)**		
No	1550(85.0)	146(81.1)
Yes	274(15.0)	28(15.6)
NA	0(0.0)	6(3.3)
**30-day mortality, n (%)**	56(3.1)	15(8.3)

[a] as the diagnoses groups and procedure groups have lengthy text details, we have not provided them in the table; they can be found in Supplementary Table 5.

[b] Definitions of variables are given in Supplementary Table 5

For peer review only

Supplementary table 5. Glossary of variables included in the PRAIS2 model

Diagnoses<br><br>Group	
1	HLHS; Truncus arteriosus; Pulmonary atresia and IVS
2	Functionally UVH; Pulmonary atresia and VSD
3	TGA þ VSD/DORV-TGA; Interrupted aortic arch
4	PDA
5	Miscellaneous primary congenital diagnosis; Tricuspid valve abnormality (including Ebstein's); TAPVC; No diagnosis given
6	Acquire
7	AVSD; Fallot/DORV Fallot
8	Aortic valve stenosis (isolated); Mitral valve abnormality; Miscellaneous congenital terms
9	TGA + IVS
10	Aortic arch obstruction þ VSD/ASD; Pulmonary stenosis
11	Subaortic stenosis (isolated); Aortic regurgitation; VSD; ASD; Arrhythmia
Procedure<br><br>diagnose group	

For peer review

1	Norwood Procedure (Stage 1); HLHS hybrid approach
2	TAPVC repair + arterial shunt; Truncus and interruption repair; Truncus arteriosus repair; Interrupted aortic arch repair; Arterial switch + aortic arch obstruction repair (with-without VSD closure)
3	Arterial shunt
4	Repair of total anomalous pulmonary venous connection; Arterial switch + VSD closure; Isolated pulmonary artery band
5	PDA ligation (surgical)
6	Arterial switch (for isolated transposition); Isolated coarctation/hypoplastic aortic arch repair; Aortopulmonary window repair
7	Senning or Mustard procedure; Ross-Konno procedure; Mitral valve replacement; Pulmonary vein stenosis procedure; Pulmonary atresia VSD repair; Tetralogy with absent pulmonary valve repair; Unifocalization procedure (with/without shunt)
8	Heart transplant; Tricuspid valve replacement; Aortic valve repair; Pulmonary valve replacement; Aortic root replacement (not Ross);Cardiac conduit replacement; Isolated RV to PA conduit construction; Tricuspid valve repair
9	Multiple VSD closure; Atrioventricular septal defect and tetralogy repair; Cor triatriatum repair; Supravalvar aortic stenosis repair; Rastelli-REV procedure
10	Bidirectional cavopulmonary shunt
11	Atrioventricular septal defect (complete) repair
12	Fontan procedure
13	Aortic valve replacement: Ross; Subvalvar aortic stenosis repair; Mitral valve repair; Sinus venosus ASD and/or PAPVC repair
14	Atrioventricular septal defect (partial) repair; Tetralogy of Fallot–type DORV repair; Vascular ring procedure
15	Anomalous coronary artery repair; Aortic valve replacement: non-Ross; ASD repair; VSD repair
20	Cardiac no specific procedure

For peer review only

Acquired Comorbidity	Pulmonary embolism; Secondary systemic hypertension; Systemic hypertension; Primary (essential) systemic hypertension; Systemic hypertension due to aortic arch obstruction; Persistent pulmonary hypertension of the new-born; Necrotising enterocolitis; Meconium aspiration; Pre-procedural coagulation disorder; Pre-procedural renal failure or Pre-procedural renal failure requiring dialysis; Pre-procedural septicaemia; Pre-procedural neurological impairment; Preprocedural cerebral abnormality on imaging; Pre-procedural tracheostomy; Preprocedural seizures; Preprocedural respiratory syncytial virus (RSV) infection; Preprocedural necrotising enterocolitis: treated medically or surgically; Psychomotor developmental delay; Brain Abscess; Cerebrovascular accident (stroke); Anoxic-ischaemic encephalopathy; Hyperthyroidism; Diabetes mellitus or Diabetes mellitus requiring insulin; Meningitis; Kidney failure; Empyema; Lower respiratory tract infection; Lung disease; Asthma; Acquired bronchial disease; Airway disease; Diaphragm disorder: acquired; Diaphragm paralysis; Oesophageal disorder
Additional cardiac risk factor	Ventricular dyssynchrony; Arrhythmogenic right ventricular cardiomyopathy; Right or Left ventricular dysfunction; Ventricular myocardial noncompaction cardiomyopathy; Infectious myocarditis; Drug induced heart muscle disease; Trypanosomal myocarditis (Chagas' disease); Myocardial failure in end stage congenital heart disease; Heart muscle disease in cardiac rejection; Nutritional heart muscle disease; Heart muscle disease in infant of diabetic mother; Heart muscle disease in collagen vascular/ connective tissue disorder; Myocarditis; Ischaemic heart disease; Cardiomyopathy; Restrictive cardiomyopathy; Idiopathic restrictive cardiomyopathy; Endocardial fibroelastosis; Infiltrative cardiomyopathy; Hypertrophic cardiomyopathy; Dilated cardiomyopathy; Pulmonary arterial hypertension; Idiopathic (primary) pulmonary hypertension; Pulmonary vascular disease; Irreversible pulmonary vascular disease due to congenital heart disease (Eisenmenger Syndrome); Secondary pulmonary hypertension; Pulmonary hypertension due to congenital systemic-to-pulmonary shunt; Elevated lung resistance for biventricular repair (> 6 Wood units); Elevated lung resistance for heart transplant (> 4 Wood units); Elevated lung resistance for univentricular repair (> 2 Wood units); Transient myocardial ischaemia; Myocardial infarction; Acute myocardial infarction; Pre-procedural pulmonary hypertension; Preprocedural myocardial infarction; Preprocedural pulmonary hypertension (pulmonary pressure more than or equal to systemic pressure): echo data or catheter data; Residual pulmonary hypertension after relief of L to R shunt

For peer review only

Congenital comorbidity	Visceral heterotaxy (abnormal arrangement thoraco-abdominal organs); Position or morphology of thoraco-abdominal organs abnormal; Lung anomaly; Functionally congenital single lung; Tracheobronchial anomaly; Intestines malrotated; Hereditary disorder associated with heart disease; Trisomy 18 - Edwards syndrome; Trisomy 13 - Pataus syndrome; 45XO - Turners syndrome; 22q11 microdeletion - CATCH 22; Syndrome/association with cardiac involvement; DiGeorge sequence; Friedreich's ataxia; Marfan syndrome; Noonan syndrome; Pompe's disease: glycogen storage disease type IIa; Tuberous sclerosis; Williams syndrome (infantile hypercalcaemia); Fetal rubella syndrome; Duchenne's muscular dystrophy; Muscular dystrophy; Ehlers-Danlos syndrome; Alagille syndrome: arteriohepatic dysplasia; Non-cardiac abnormality associated with heart disease; Non-cardiothoracic / vascular abnormality (DESCRIBE); Cystic fibrosis; Diaphragmatic hernia; Tracheo-oesophageal fistula; Omphalocoele; Duodenal stenosis/atresia; Sickle cell disease; Renal abnormality; Congenital coagulation disorder; Thoracic / mediastinal abnormality; Microcephaly; Choanal atresia; Tracheobronchial malacia; Hypothyroidism; Cerebral anomaly; Connective tissue disease; Kyphoscoliosis; Cleft lip / palate; Loeys-Dietz Syndrome (transforming growth factor beta receptor (TGFBR) gene); Von Willebrand disease; Maternally derived fetal disease or syndrome associated with heart disease; Major anomaly of gastrointestinal system; Multiple congenital malformations; Tracheal stenosis or disease
Severity of illness	Pre-procedural shock; Pre-procedural acidosis; Pre-procedural mechanical ventilatory or circulatory support; Preprocedural shock at time of surgery (persistent); Preprocedural cardiopulmonary resuscitation (< 48 hours); Cardiac Arrest; Respiratory failure;

ASD = atrial septal defect; AVSD = atrioventricular septal defect; DORV = double outlet right ventricle; HLHS = hypoplastic left heart syndrome; IVS = intact ventricular septum; PA = pulmonary artery; PAPVC = partial anomalous pulmonary venous connection; PDA = patent ductus arteriosus; TAPVC = total anomalous pulmonary venous connection; REV = reparation a letage ventriculaire; RV = right ventricle; TGA = transposition of the great arteries; UVH = univentricular heart; VSD = ventricular septal defect

For peer review only

For peer review only

6a. Hosmer and Lemeshow goodness of fit groups, external validation cohort 2

PRAIS2 categories	Predicted survival	Predicted mortality	Observed survival	Observed mortality
[0.000131,0.00126]	119.93	0.07	120.00	0.00
(0.00126,0.00277]	119.76	0.24	120.00	0.00
(0.00277,0.00496]	118.54	0.46	119.00	0.00
(0.00496,0.0071]	119.28	0.72	120.00	0.00
(0.0071,0.0101]	118.97	1.03	119.00	1.00
(0.0101,0.0145]	117.59	1.41	118.00	1.00
(0.0145,0.0206]	117.94	2.06	120.00	0.00
(0.0206,0.0338]	115.78	3.22	114.00	5.00
(0.0338,0.0743]	114.04	5.96	117.00	3.00
(0.0743,0.408]	102.32	17.68	107.00	13.00

Supplementary Table 6b Hosmer and Lemeshow goodness of fit groups, external validation cohort 1

PRAIS2 categories	Predicted survival	Predicted mortality	Observed survival	Observed mortality
[3.61e-05,0.000938]	135.94	0.06	136.00	0.00
(0.000938,0.00249]	134.77	0.23	134.00	1.00
(0.00249,0.00482]	134.51	0.49	135.00	0.00
(0.00482,0.00803]	134.12	0.88	133.00	2.00
(0.00803,0.0115]	133.69	1.31	131.00	4.00
(0.0115,0.0163]	133.16	1.84	133.00	2.00
(0.0163,0.0221]	132.46	2.54	133.00	2.00
(0.0221,0.0305]	131.49	3.51	128.00	7.00
(0.0305,0.046]	130.01	4.99	127.00	8.00
(0.046,0.256]	124.00	12.00	130.00	6.00

For peer review only

6c. Hosmer and Lemeshow goodness of fit groups, overlapping cohorts

PRAIS2 categories	Predicted survival	Predicted mortality	Observed survival	Observed mortality
[4.29e-05,0.000909]	182.92	0.08	183.00	0.00
(0.000909,0.00234]	181.72	0.28	181.00	1.00
(0.00234,0.0045]	181.39	0.61	180.00	2.00
(0.0045,0.00754]	181.91	1.09	181.00	2.00
(0.00754,0.0108]	180.35	1.65	181.00	1.00
(0.0108,0.0159]	179.61	2.39	178.00	4.00
(0.0159,0.0229]	179.48	3.52	182.00	1.00
(0.0229,0.0327]	177.03	4.97	176.00	6.00
(0.0327,0.0539]	174.60	7.40	171.00	11.00
(0.0539,0.351]	164.34	18.66	155.00	28.00

6d. Hosmer and Lemeshow goodness of fit groups, in non-elective subset

PRAIS2 categories	Predicted survival	Predicted mortality	Observed survival	Observed mortality
[0.00109,0.00594]	48.82	0.18	49.00	0.00
(0.00594,0.00947]	47.64	0.36	48.00	0.00
(0.00947,0.0138]	47.46	0.54	47.00	1.00

(0.0138,0.0201]	47.20	0.80	48.00	0.00
(0.0201,0.0287]	47.80	1.20	47.00	2.00
(0.0287,0.0384]	46.44	1.56	44.00	4.00
(0.0384,0.0546]	45.83	2.17	46.00	2.00
(0.0546,0.0818]	44.71	3.29	47.00	1.00
(0.0818,0.126]	43.02	4.98	46.00	2.00
(0.126,0.408]	38.42	10.58	39.00	10.00

For peer review only

Supplementary table 7. Metrics of model discrimination according to different PRAIS-2 cut-off values in the Cohort 1

	Metrics	Top 90%	Top 80%	Top 20%	Top 10%
**Cohort 1**	**Cut off**	0.0009	0.0025	0.03	0.046
	**Sensitivity**	1	0.96	0.44	0.19
	**Specificity**	0.1	0.20	0.80	0.90
	**PPV**	0.03	0.03	0.05	0.04
	**NPV**	1	0.99	0.98	0.98
	**LRP**	1.11	1.21	2.23	1.89
	**LRN**	0	0.15	0.69	0.90

LRP= positive--likelihood ratios; LRN= negative-likelihood ratios; PPV= positive predictive values; NPV=negative predictive values

.

For peer review only

TRIPOD Checklist: Prediction Model Validation

Section/Topic	Item	Checklist Item	Page
**Title and abstract**			
Title	1	Identify the study as developing and/or validating a multivariable prediction model, the target population, and the outcome to be predicted.	1
Abstract	2	Provide a summary of objectives, study design, setting, participants, sample size, predictors, outcome, statistical analysis, results, and conclusions.	2
**Introduction**			
Background and objectives	3a	Explain the medical context (including whether diagnostic or prognostic) and rationale for developing or validating the multivariable prediction model, including references to existing models.	4-5
	3b	Specify the objectives, including whether the study describes the development or validation of the model or both.	4-5
**Methods**			
Source of data	4a	Describe the study design or source of data (e.g., randomized trial, cohort, or registry data), separately for the development and validation data sets, if applicable.	6-7
	4b	Specify the key study dates, including start of accrual; end of accrual; and, if applicable, end of follow-up.	6-7
Participants	5a	Specify key elements of the study setting (e.g., primary care, secondary care, general population) including number and location of centres.	6-7
	5b	Describe eligibility criteria for participants.	6-7
	5c	Give details of treatments received, if relevant.	6-7
Outcome	6a	Clearly define the outcome that is predicted by the prediction model, including how and when assessed.	10
	6b	Report any actions to blind assessment of the outcome to be predicted.	NA
Predictors	7a	Clearly define all predictors used in developing or validating the multivariable prediction model, including how and when they were measured.	9
	7b	Report any actions to blind assessment of predictors for the outcome and other predictors.	NA
Sample size	8	Explain how the study size was arrived at.	6-8
Missing data	9	Describe how missing data were handled (e.g., complete-case analysis, single imputation, multiple imputation) with details of any imputation method.	8
Statistical analysis methods	10c	For validation, describe how the predictions were calculated.	9
	10d	Specify all measures used to assess model performance and, if relevant, to compare multiple models.	10
	10e	Describe any model updating (e.g., recalibration) arising from the validation, if done.	NA
Risk groups	11	Provide details on how risk groups were created, if done.	7-8
Development vs. validation	12	For validation, identify any differences from the development data in setting, eligibility criteria, outcome, and predictors.	12-13
**Results**			
Participants	13a	Describe the flow of participants through the study, including the number of participants with and without the outcome and, if applicable, a summary of the follow-up time. A diagram may be helpful.	5-6
	13b	Describe the characteristics of the participants (basic demographics, clinical features, available predictors), including the number of participants with missing data for predictors and outcome.	8
	13c	For validation, show a comparison with the development data of the distribution of important variables (demographics, predictors and outcome).	9
Model performance	16	Report performance measures (with CIs) for the prediction model.	10
Model-updating	17	If done, report the results from any model updating (i.e., model specification, model performance).	NA
**Discussion**			
Limitations	18	Discuss any limitations of the study (such as nonrepresentative sample, few events per predictor, missing data).	16-18
Interpretation	19a	For validation, discuss the results with reference to performance in the development data, and any other validation data.	16-18
	19b	Give an overall interpretation of the results, considering objectives, limitations, results from similar studies, and other relevant evidence.	16-18
Implications	20	Discuss the potential clinical use of the model and implications for future research.	16-18
**Other information**			
Supplementary information	21	Provide information about the availability of supplementary resources, such as study protocol, Web calculator, and data sets.	8,9,13,14,15,18
Funding	22	Give the source of funding and the role of the funders for the present study.	18,23

We recommend using the TRIPOD Checklist in conjunction with the TRIPOD Explanation and Elaboration document.

TRIPOD Checklist: Prediction Model Validation

For peer review only

TRIPOD Checklist: Prediction Model Validation

	Page
Title	1
Abstract	2
Strengths and limitations of this study	3
Introduction	4-5
Methods	6-10
Results	11-15
Discussion	15-18
Acknowledge	18
Funding	18
Conflict	19
Figure legend	20
References	21-22
Contributorship statement	23

For peer review only
